# The Tumor Suppressor CYLD Inhibits Mammary Epithelial to Mesenchymal Transition by the Coordinated Inhibition of YAP/TAZ and TGFβ Signaling

**DOI:** 10.3390/cancers12082047

**Published:** 2020-07-24

**Authors:** Athanasios Pseftogas, Konstantinos Xanthopoulos, Theofilos Poutahidis, Chrysanthi Ainali, Dimitra Dafou, Emmanuel Panteris, Joseph G. Kern, Xaralabos Varelas, Alexander Hardas, Christos Gonidas, Anastasia Tsingotjidou, Eudoxia Hatzivassiliou, George Mosialos

**Affiliations:** 1 School of Biology, Aristotle University of Thessaloniki, 54124 Thessaloniki, Greece; apseftog@bio.auth.gr (A.P.); xantho@pharm.auth.gr (K.X.); dafoud@bio.auth.gr (D.D.); epanter@bio.auth.gr (E.P.); cgonidasp@bio.auth.gr (C.G.); 2 Division of Experimental Oncology, IRCCS Istituto Scientfico San Raffaele, 20132, Milan, Italy; 3 School of Pharmacy, Aristotle University of Thessaloniki, 54124 Thessaloniki, Greece; 4 School of Veterinary Medicine, Aristotle University of Thessaloniki, 54124 Thessaloniki, Greece; teoput@vet.auth.gr (T.P.); achardas@rvc.ac.uk (A.H.); astsing@vet.auth.gr (A.T.); 5 St John’s Institute of Dermatology, School of Basic and Medical Biosciences, King’s College London, London SE1 9RT, UK; chrysanthi.ainali@kcl.ac.uk; 6 Department of Biochemistry, Boston University School of Medicine, Boston, MA 02118, USA; jkern@bu.edu (J.G.K.); xvarelas@bu.edu (X.V.); 7 Department of Pathobiology & Population Sciences, The Royal Veterinary College, Hawkshead Lane, North Mymms, Hatfield, Hertfordshire AL9 7TA, UK; 8 School of Medicine, Aristotle University of Thessaloniki, 54124 Thessaloniki, Greece; eudoxiah@auth.gr

**Keywords:** breast cancer, EMT, TGFβ, YAP, TAZ, CYLD

## Abstract

Downregulation of the cylindromatosis (CYLD) tumor suppressor has been associated with breast cancer development and progression. Here, we report a critical role for CYLD in maintaining the phenotype of mammary epithelial cells in vitro and in vivo. CYLD downregulation or inactivation induced an epithelial to mesenchymal transition of mammary epithelial cells that was dependent on the concomitant activation of the transcription factors Yes-associated protein (YAP)/transcriptional coactivator with PDZ-binding motif (TAZ) and transforming growth factor beta (TGFβ)signaling. CYLD inactivation enhanced the nuclear localization of YAP/TAZ and the phosphorylation of Small Mothers Against Decapentaplegic (SMAD)2/3 proteins in confluent cell culture conditions. Consistent with these findings were the hyperplastic alterations of CYLD-deficient mouse mammary epithelia, which were associated with enhanced nuclear expression of the YAP/TAZ transcription factors. Furthermore, in human breast cancer samples, downregulation of CYLD expression correlates with enhanced YAP/TAZ-regulated target gene expression. Our results identify CYLD as a critical regulator of a signaling node that prevents the coordinated activation of YAP/TAZ and the TGFβ pathway in mammary epithelial cells, in order to maintain their phenotypic identity and homeostasis. Consequently, they provide a novel conceptual framework that supports and explains a causal implication of deficient CYLD expression in aggressive human breast cancers.

## 1. Introduction

The epithelial to mesenchymal transition (EMT) has been recognized as an important mechanism that can promote invasion and metastasis of breast cancer cells (reviewed in [1]). EMT represents a dedifferentiation process of epithelial cells that can generate developmentally primitive states, which are associated with aggressive behavior. Indeed, EMT in breast cancer has been associated with the development of the cancer stem cell state and chemoresistance. Although several signaling pathways have been implicated in the induction of EMT in breast cancer, the molecular mechanisms that can trigger the deregulation of these pathways, and induce EMT have not been identified comprehensively.

Inactivating mutations of the *CYLD* gene or its downregulated expression have been documented in several types of human tumors [2,3,4,5,6,7,8,9,10,11,12]. The tumor-suppressing function of *CYLD* is also supported by the increased oncogenic susceptibility of *CYLD*-deficient mouse models to proinflammatory and/or genotoxic stress [13,14,15,16,17,18]. The human *CYLD* gene is expressed in most tissues and codes for a 956-amino-acid deubiquitinating enzyme (CYLD), which selectively hydrolyzes K63- and M1-linked polyubiquitin chains [2,19]. The deubiquitinating domain of CYLD is located at the carboxyl-terminal region of the protein and three CAP-Gly domains are found within the CYLD amino terminal region, two of which are capable of interacting with microtubules and their associated proteins end-binding protein 1 (EB1) and histone deacetylase 6 (HDAC6) [20]. These interactions have been implicated in the regulation of microtubule dynamics by CYLD, which affect cell migration and various aspects of the cell cycle [21,22,23]. CYLD has been implicated in the regulation of various signaling pathways that affect cell proliferation and survival. Its role in the signal transduction by tumor necrosis factor receptor 1 (TNFR1) has been studied extensively. CYLD is capable of inhibiting TNFR1-mediated Nuclear Factor-kappaB (NF-kappaB) and c-Jun N-terminal kinase (JNK) activation by hydrolyzing K63- and M1-linked polyubiquitin scaffolds that are assembled in response to receptor activation on various signaling components [24,25,26]. CYLD also plays an important role in the promotion of necroptosis by TNFR1 by the deubiquitination of Receptor-interacting serine/threonine-protein kinase 1(RIPK1), which facilitates the assembly of an active RIPK1-RIPK3 complex [27]. It should be noted that the effect of CYLD on various signaling processes can be cell-type specific. Therefore, it is important to assess and characterize its role in different cell types at the molecular level in order to understand its involvement in mammalian pathophysiology.

Multiple lines of evidence from cell line models and human patient samples have implicated CYLD in breast cancer suppression [28,29,30,31,32,33,34,35]. Downregulation of *CYLD* expression can augment the viability, migratory capacity, and anchorage-independent growth of basal and luminal human breast cancer cell lines [28,29,30,31,33,34,35]. In addition, CYLD protein downregulation was correlated with poor prognosis in primary breast cancer patients [35]. Upregulation of NF-kappaB or JNK activities was observed in specific cases of CYLD-deficient breast cancer cell lines. However, a comprehensive understanding of the molecular and cellular mechanisms that underly the role of CYLD in mammary epithelia homeostasis has not been established.

In the present report, we evaluated the phenotypic effects of CYLD inactivation or downregulation in non-transformed mammary epithelial cells. Our experiments identified a novel role for CYLD in inhibiting mammary epithelial to mesenchymal transition (EMT) through the coordinated downregulation of YAP/TAZ and TGFβ signaling pathways.

## 2. Results

### 2.1. Downregulation of CYLD in Mammary Epithelial Cells Promotes Mesenchymal Phenotypic Characteristics

**Figure 1:**
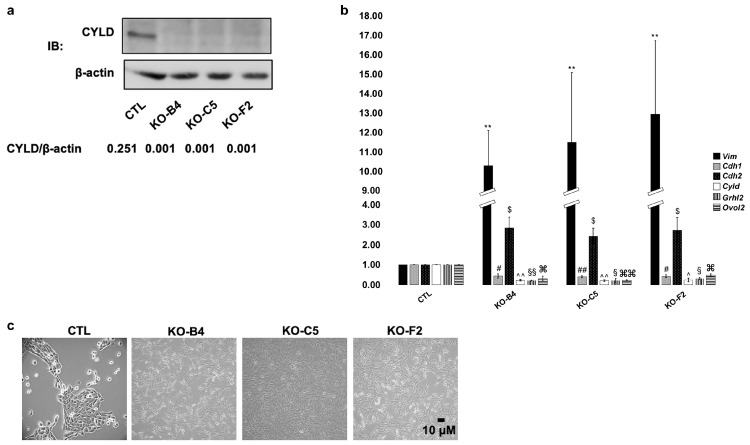
Introduction of CYLD-inactivating mutations in MCF10A cells induces EMT-like changes. (**a**) MCF10A cells were infected with lentiviral vectors expressing Cas9 and gRNAs that target the GFP gene (control gene) or exons two, three, or nine of the *CYLD* gene. Whole cell lysates extracted from control (CTL) and CYLD-targeted (B4, C5, and F2) clones were isolated and analyzed by immunoblotting for the expression of CYLD and β-actin. The indicated ratios of band intensities are shown below the corresponding lane. (**b**) Genetic inactivation of CYLD leads to reduction of *E-cadherin* (Cdh1), *Grainyhead Like Transcription Factor 2* (Grhl2), and *Ovo Like Zinc Finger 2* (Ovol2), and upregulation of *Vimentin* (Vim) and *N-cadherin* (Cdh2) mRNA expression levels. Total RNA was extracted from the MCF10A clones analyzed in A and used to determine the relative levels of the indicated mRNAs by qPCR. The histogram indicates the average values (+/− SE) of relative mRNA levels as determined by the ΔΔC_T_ method and *tyrosine 3-monooxygenase/tryptophan 5-monooxygenase activation protein zeta* (*YWHAZ*) as the endogenous control from at least three independent experiments. The statistical analysis of relative mRNA expression pairwise comparisons between the control clone and each one of the CYLD-deficient clones was performed by the Student’s *t*-test method. ($, #, ^, §, ⌘: *p* ≤ 0.05, **, ##, ^^, §§, ⌘⌘: *p* ≤ 0.01). (**c**) Representative pictures of the MCF10A clones analyzed in (**a**).

The effects of CYLD deficiency and overexpression in breast cancer cell lines have suggested important tumor suppressive roles [33,34,35]. To gain insight into the tumor suppressive roles of CYLD in mammary epithelium, we used CRISPR/Cas9 to generate clones of non-transformed MCF10A cells that possess different mutations in the CYLD gene, which result in the loss of full-length CYLD protein (Figure 1a) and reduction of CYLD mRNA expression (Figure 1b) that is likely due to non-sense mRNA decay (NMD). NMD is a mechanism of mRNA degradation when premature stop codons are introduced [36]. As shown in Figure 1c, all three MCF10A clones with protein-truncating *CYLD* mutations showed dramatic morphological changes that involved reduced cell–cell contacts and an elongated shape. Consistent with the morphological changes, it was noted that cells with mutated CYLD had reduced mRNA expression of the epithelial marker E-cadherin and increased expression of mesenchymal markers vimentin and N-cadherin compared to control cells (Figure 1b), which together suggested that CYLD-deficient cells gained mesenchymal traits. The elevated levels of N-cadherin expression in CYLD-deficient cells were also documented by immunoblotting (Figure S1).

In order to exclude the possibility that the observed changes were due to clonal or off-target CRISPR/Cas9 effects, the expression of CYLD was downregulated by RNA interference in MCF10A cells. A substantial fraction of the cells that were transfected with a *CYLD*-targeting siRNA showed striking morphological changes that were manifested as a loss of extensive contacts with neighboring cells and the acquisition of an elongated spindle-like shape, similarly to the results obtained with CRISPR/Cas9-mediated inactivation of CYLD (Figure S2a). Cells transfected with *CYLD*-targeting siRNA also showed reduced expression of the epithelial marker *E-cadherin* and increased expression of the mesenchymal markers *vimentin* and *N-cadherin* compared to control siRNA-transfected cells (Figure S2b). Examination of the expression level of transcription factors that are known to mediate the establishment of a mesenchymal phenotype demonstrated that reduced CYLD expression caused a significant increase in the expression of *Snail1* and *Zinc finger E-box-binding homeobox 2* (ZEB2) (Figure S2c). Immunofluorescence analysis confirmed the reduction in the levels of E-cadherin protein and the increase in the levels of vimentin in cells with mesenchymal morphology (Figure S2d). These changes were not specific to MCF10A cells since similar changes were also observed in MCF7 cells that were subjected to CYLD downregulation by RNA interference (Figure S3). These findings indicate that CYLD deficiency in mammary epithelial cells is sufficient to induce morphological and gene expression changes that are consistent with the acquisition of a mesenchymal phenotype.

### 2.2. CYLD Deficiency Impairs the Proper Development and Organization of Mammary Spheroids

**Figure 2:**
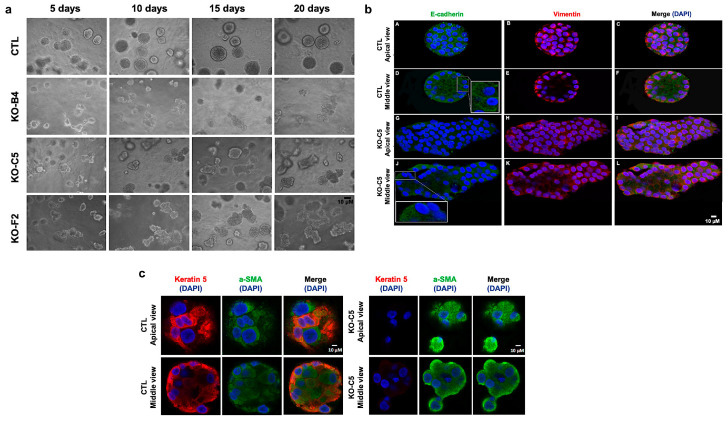
CYLD inactivation compromises the proper development of MCF10A mammospheres. (**a**) Control (CTL) and CYLD-deficient MCF10A cells (KO-B4, KO-C5, and KO-F2) were cultured in the presence of Matrigel for 20 days and photographed. (**b**) Expression pattern of E-cadherin (green) and vimentin (red) in control and CYLD-deficient MCF10A clones cultured in the presence of Matrigel for 20 days. The cell nuclei were stained with 4′,6-diamidino-2-phenylindole (DAPI) (blue). Inset images represent 200× magnification of the corresponding areas. (**c**) Expression pattern of keratin-5 (red) and smooth muscle actin (α-SMA, green) in control and CYLD-deficient MCF10A clones cultured in the presence of Matrigel for 20 days. The cell nuclei were stained with DAPI (blue). Representative data from one out of two independent experiments are shown.

Culture of MCF10A epithelial cells under the proper conditions in semisolid media gives rise to mammary spheroids that have the characteristic luminal cavity. In order to determine whether CYLD deficiency affects the capacity of MCF10A cells to form properly organized spheroids, control and CYLD-deficient clones of MCF10A cells were cultured in Matrigel-containing media, as described in the materials and methods. As shown in Figure 2, control MCF10A cells formed well-rounded spheroids with a distinct luminal area and the proper cortical distribution of E-cadherin. Strikingly, CYLD-deficient MCF10A cells formed mainly structures of an irregular shape and luminal structure. In the CYLD-deficient cultures, E-cadherin was localized mainly in the cytoplasm, whereas cortical E-cadherin was more prominent in control spheroids. Furthermore, a small but statistically significant increase in the expression level of vimentin was detected in CYLD-deficient spheroids compared to control ones (Figure 2b and Figure S4). Finally, CYLD-deficient spheroids lacked keratin-5 expression and had a higher expression of smooth muscle actin (Figure 2c and Figure S4). These findings are consistent with a critical role for CYLD in the maintenance of the phenotypic characteristics of mammary epithelial cells when grown in three dimensions.

### 2.3. Inactivation of CYLD in Mammary Epithelial Cells Promotes the Development of Stem Cell and Tumorigenic Characteristics

**Figure 3:**
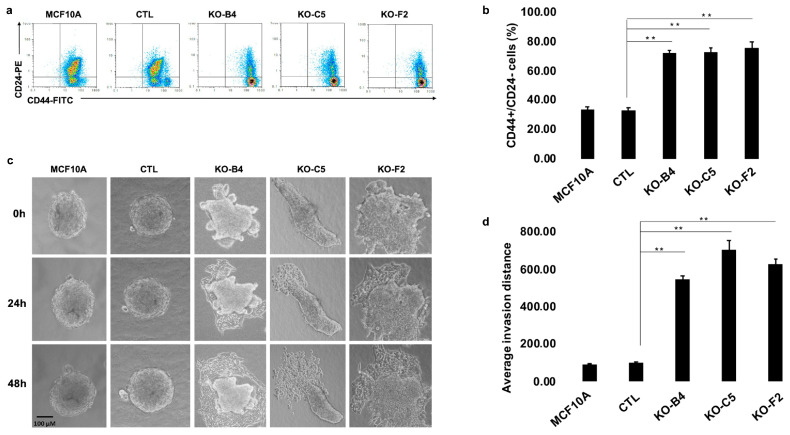
CYLD inactivation promotes the development of stem cell and invasive phenotypic characteristics. (**a**) Representative flow cytometric detection of CD44 and CD24 in control (MCF10A, CTL) and CYLD-deficient MCF10A (KO-B4, KO-C5, and KO-F2) cells for the expression of CD44 and CD24 markers. (**b**) Relative representation of CD44+/CD24− cells in control (MCF10A, CTL) and CYLD-deficient MCF10A (KO-B4, KO-C5, and KO-F2) cells. Average values (+/− SE) from three independent experiments are shown (** *p* ≤ 0.01). (**c**) Representative images of a three-dimensional invasion assay from control (MCF10A, CTL) and CYLD-deficient MCF10A (KO-B4, KO-C5, and KO-F2) cells at time points of 0, 24, and 48 h. (**d**) Representation of the average invasion distance of CYLD-deficient MCF10A (KO-B4, KO-C5, and KO-F2) cells. Average values (+/− SE) from three independent experiments are shown (** *p* ≤ 0.01).

The EMT of mammary epithelial cells has been recognized as a process that can promote the appearance of cancer stem cell markers [37]. In order to determine whether the inactivation of CYLD in MCF10A cells can confer such attributes to these cells, the expression of CD44 and CD24 markers was evaluated. As shown in Figure 3a, b, CYLD-deficient cells demonstrated a higher expression of CD44 and a reduced expression of CD24 compared to control cells. These changes were also reflected in the mRNA levels of *CD44* and *CD24* (Figure S5). This profile is a characteristic feature of breast cancer cells with stem-like properties [38,39]. In order to evaluate the invasive properties of control and CYLD-deficient MCF10A cells, the cells were cultured in the appropriate matrix and photographed at 24, 48, and 72 h. Cells that lacked functional CYLD formed well-defined monolayers invading into the surrounding extracellular matrix (Figure 3c,d), a phenotype that was not observed in the cultures of control cells. These findings indicate that loss of functional CYLD in mammary epithelial cells confers stem-like and invasive properties, which are consistent with the aggressive behavior of CYLD-deficient human breast cancers.

### 2.4. Inactivation of CYLD in Mammary Epithelial Cells Coordinately Activates the TGFβ and YAP/TAZ Pathways

**Figure 4:**
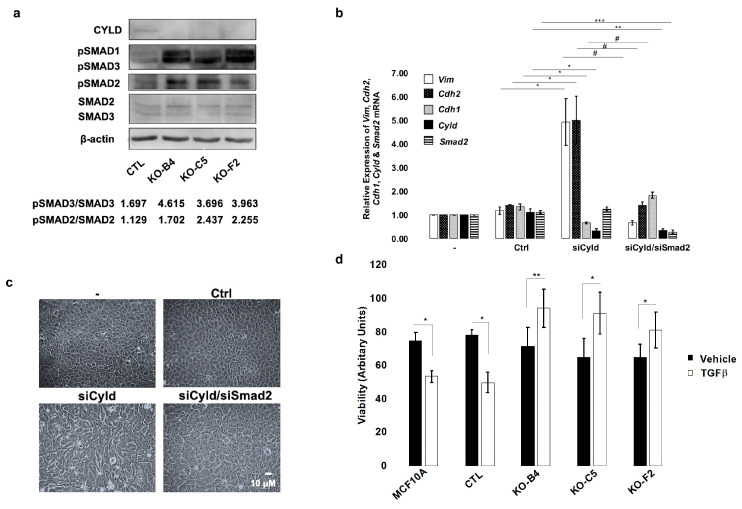
CYLD-regulated mammary EMT depends on the activation of the TGFβ signaling pathway. (**a**) Immunoblot analysis of CYLD pSMAD1, pSMAD2, pSMAD3, SMAD2, SMAD3, and β-actin expression in whole cell extracts from control (CTL) and CYLD-deficient MCF10A cells (KO-B4, KO-C5, and KO-F2). The indicated ratios of band intensities are shown below the corresponding lane. (**b**) Simultaneous *CYLD* and *SMAD2* downregulation inhibits the EMT process that is induced by *CYLD* downregulation. MCF10A cells were transfected with *CYLD*-targeting (siCyld), *SMAD2*-targeting (siSmad2), or luciferase-targeting (Ctrl) siRNAs as indicated. After 48 h, total RNA was extracted and used to determine the relative levels of the indicated *vimentin* (Vim), *E-cadherin* (Cdh1), *N-cadherin* (Cdh2), *CYLD* (Cyld), and *SMAD2* (Smad2) mRNAs by qPCR. The histogram indicates the average values (+/− SE) of relative mRNA levels as determined by the ΔΔC_T_ method using *YWHAZ* as the endogenous control, from at least three independent experiments. The statistical analysis of the pairwise comparisons indicated by horizontal lines was performed by the Student’s *t*-test method. (*, #: *p* ≤ 0.05, ** *p* ≤ 0.01, *** *p* ≤ 0.001). (**c**) Pictures of cells analyzed in (**b**). (**d**) Control (MCF10A, CTL) and CYLD-deficient MCF10A cells (KO-B4, KO-C5, and KO-F2) were grown in the presence or absence of TGFβ for 24 h and quantitated. Average values (+/− SE) of cell viability relative to the initiation of treatment from three independent experiments are shown. The statistical analysis of the pairwise comparisons indicated by brackets was performed by the Student’s *t*-test method. (* *p* ≤ 0.05, ** *p* ≤ 0.01).

Signaling induced by the TGFβ growth factor is well-established as an inducer of EMT [40]. We therefore investigated whether CYLD-deficient MCF10A cells exhibited TGFβ pathway activation characteristics. Interestingly, CYLD-deficient cells showed a significantly increased basal phosphorylation of SMAD2 and SMAD3, compared to control cells (Figure 4a). The protein levels of SMAD2 and SMAD3 were unaltered in CYLD-deficient cells, suggesting spontaneous activation of the TGFβ pathway. In order to determine whether the activation of the TGFβ pathway was essential for the EMT changes that were observed in CYLD-deficient cells, MCF10A cells were transfected with a *CYLD*-downregulating siRNA in the absence and presence of siRNA targeting *SMAD2*. As shown in Figure 4b, the expression of *CYLD* and *SMAD2* mRNAs was successfully downregulated by the respective siRNAs. As expected, cells with downregulated CYLD expression showed increased expression of the mesenchymal markers *vimentin* and *N-cadherin* and downregulated expression of the epithelial marker *E-cadherin*. Interestingly, the concomitant downregulation of SMAD2 prevented the acquisition of mesenchymal phenotypic characteristics by CYLD-deficient cells. This was evident in cell morphology (Figure 4c) and in the expression profile of *vimentin*, *N-cadherin*, and *E-cadherin* (Figure 4b). These findings indicate that the activation of TGFβ-SMAD2 signaling is an essential mechanism for the mesenchymal transition that is induced by CYLD deficiencies.

TGFβ pathway signaling has complex roles in carcinogenesis with cytostatic effects in early stages and protumorigenic roles that include the promotion of EMT in late metastatic stages. Despite exhibiting increased TGFβ pathway activity, the CYLD-deficient MCF10A cell lines showed similar apparent growth characteristics to their parental counterparts, suggesting that CYLD inactivation may counteract the cytostatic effect of the TGFβ pathway. To directly test this, control and CYLD-deficient MCF10A cells were incubated in the absence or presence of exogenous TGFβ for various time points and their viability was determined. CYLD-deficient MCF10A cells were insensitive to the cytostatic effect of TGFβ, as demonstrated by their increased viability, which was significantly reduced in the parental MCF10A cells (Figure 4d).

**Figure 5:**
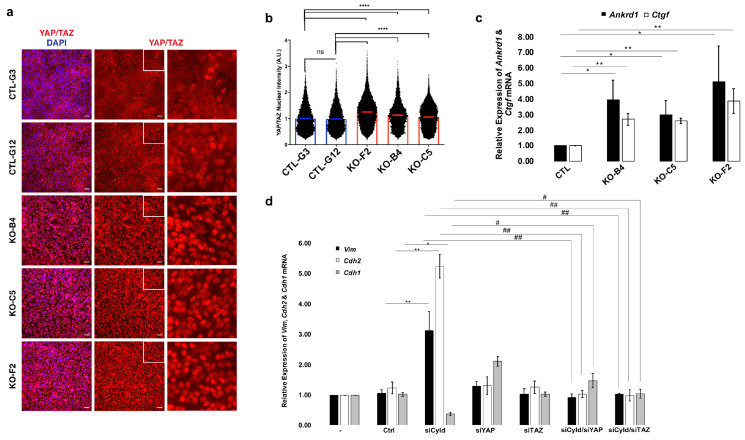
CYLD-regulated mammary EMT depends on the inhibition of the Hippo pathway. (**a**) Immunofluorescence analysis of YAP and TAZ localization in control (CTL-) and CYLD-deficient MCF10A clones (KO-). (**b**) YAP/TAZ staining intensity in individual nuclei was measured relative to the average nuclear area from panel **a**. Results were normalized to clone CTL-G3 to obtain fold changes in YAP/TAZ levels between clones. Statistical analysis was performed using one-way ANOVA and Tukey’s multiple comparisons tests. CTL-G3 (*n* = 7926), CTL-G12 (*n* = 6778), KO-F2 (*n* = 14360), KO-B4 (*n* = 5883), and KO-C5 (*n* = 8453). Error bars represent mean +/− SD. (**** *p* < 0.0001) (**c**) CYLD inactivation leads to the induction of YAP/TAZ target genes. Total RNA was extracted from control (CTL) and CYLD-deficient MCF10A cells (KO-B4, KO-C5, and KO-F2) and used to determine the relative levels of the indicated *ANKRD1* (*Ankrd1*) and *CTGF* (*Ctgf*) mRNAs using qPCR. The histogram indicates the average values (+/− SE) of relative mRNA levels as determined by the ΔΔC_T_ method using *YWHAZ* as the endogenous control from at least three independent experiments. The statistical analysis of the pairwise comparisons indicated by horizontal lines was performed by the Student’s *t*-test method. (* *p* ≤ 0.05, ** *p* ≤ 0.01). (**d**) Simultaneous *CYLD* and *YAP* or *TAZ* downregulation prevents the EMT process that is induced by *CYLD* downregulation. MCF10A cells were transfected with CYLD-targeting (siCyld), *YAP*-targeting (siYap), *TAZ*-targeting (siTaz), or luciferase-targeting (Ctrl) siRNAs as indicated. After 48 h, total RNA was extracted and used to determine the relative levels of the indicated *vimentin* (Vim), *E-cadherin* (Cdh1), *N-cadherin* (Cdh2), and *CYLD* (Cyld) mRNAs by qPCR. The histogram indicates the average values (+/− SE) of relative mRNA levels as determined by the ΔΔC_T_ method using *YWHAZ* as the endogenous control from at least three independent experiments. The statistical analysis of the pairwise comparisons indicated by horizontal lines and brackets was performed by the Student’s *t*-test method (*, #: *p* ≤ 0.05, **, ##: *p* ≤ 0.01).

The nuclear activation of the transcriptional effectors YAP and TAZ can counteract the cytostatic effect of TGFβ, promoting anchorage-independent growth and enhanced migratory capacity [41]. YAP/TAZ are key effectors of the Hippo signaling pathway, which are regulated in part by a phosphorylation cascade relayed by the kinases Macrophage Stimulating (MST)1/2 and Large Tumor Suppressor Kinase (LATS) 1/2 [42]. Upon Hippo pathway kinase inactivation, YAP/TAZ accumulate in the nucleus and permit enhanced nuclear SMAD activation [41,43]. We therefore tested whether loss of CYLD impacted YAP/TAZ localization and activity. We found that CYLD-deficient cells showed elevated nuclear YAP/TAZ levels (Figure 5a, b) and YAP/TAZ target genes, such as *connective tissue growth factor* (*CTGF*) and *Ankyrin Repeat Domain 1* (*ANKRD1*), showed increased expression compared to control cells (Figure 5c). The total levels of YAP or TAZ proteins did not change substantially upon inactivation of CYLD (Figure S6a). Taken together, the results shown in Figure 5c and Figure S6a support the notion of enhanced nuclear translocation of YAP and TAZ upon CYLD inactivation. To determine whether the activity of YAP and TAZ is required for the phenotypes induced by CYLD downregulation, we concomitantly downregulated *CYLD* and *YAP* or *TAZ* expression using RNA interference (Figure S6b). We found that downregulation of either *YAP* or *TAZ* prevented the EMT that is induced by *CYLD* downregulation (Figure 5d). These observations indicated that loss of CYLD leads to enhanced YAP/TAZ activity, which is essential for the observed EMT.

### 2.5. Inactivation of CYLD in the Mouse Mammary Epithelium Results in Hyperplastic Alterations

**Figure 6:**
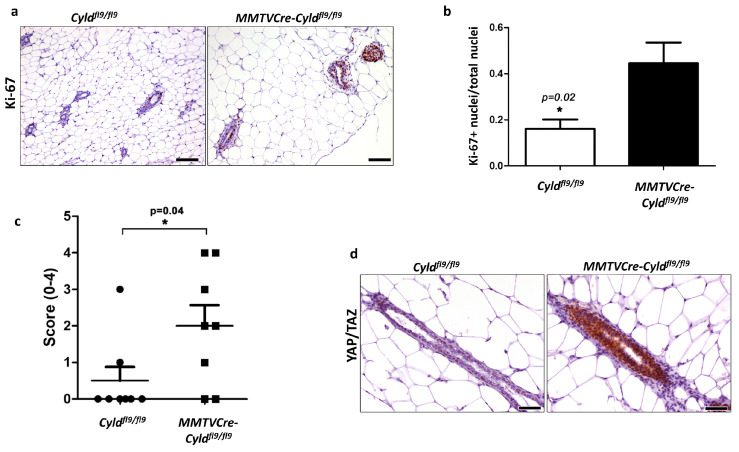
Immunohistochemical analysis of control and CYLD-deficient mouse mammary glands. (**a**) Comparison of Ki-67 expression in mammary epithelia of control (*Cyld^fl9/fl9^*) and CYLD-deficient (*MMTVCre-Cyld^fl9/fl9^*) mice. Bar = 100 μM. (**b**) Quantitation of Ki-67 expression in mammary epithelia of control (*Cyld^fl9/fl9^*) and CYLD-deficient (*MMTVCre-Cyld^fl9/fl9^*) mice. (**c**) Hyperplasia was semi-quantitatively assessed in mammary gland sections from control and CYLD-deficient mice of a matched background (C57BL/6-BALB/c (1:1)) as described in the materials and methods. (**d**) Comparison of YAP/TAZ expression in mammary epithelia of control (*Cyld^fl9/fl9^*) and CYLD-deficient (*MMTVCre-Cyld^fl9/fl9^*) mice. Bar = 50 μM. * *p* < 0.05.

To determine whether CYLD plays an important role in the homeostasis of the mammary epithelium in vivo, mice with targeted inactivation of CYLD in the mammary epithelium were generated and analyzed. Towards this goal, previously generated mice with a floxed ninth coding exon (Cyld^fl9^ mice, [44]) were crossed with mice expressing the Cre recombinase under the control of the MMTV promoter (Figure S7, [45]). The genetic background of the mice was a mixed C57BL/6-BALB/c (1:1) background. Truncation of the catalytic domain of CYLD as a result of elimination of exon 9, resulted in a high frequency of mice with hyperplastic mammary epithelia (Figure 6a–c). In contrast to the mammary tissue of control mice that showed small numbers of Ki-67-positive (proliferating) epithelial cells in selected gland profiles, mammary glands from CYLD-deficient mice showed multifocal to multifocally diffuse areas containing gland profiles with prominent Ki-67 expression (Figure 6a, b).

In addition, mammary glands from CYLD-deficient mice displayed high levels of nuclear YAP/TAZ staining, consistent with our observations in vitro (Figure 6d). We did not observe other apparent abnormalities in mice with mammary epithelial CYLD deficiency up to the age of 8 weeks, at which time point the mice were sacrificed for histological analysis. These results extend our findings from the cell line models to establish a critical role for CYLD in mammary epithelia architecture and homeostasis in vivo.

### 2.6. CYLD Downregulation in Human Breast Cancer Patient Samples Correlates with the Activation of the YAP/TAZ Pathway.

**Figure 7:**
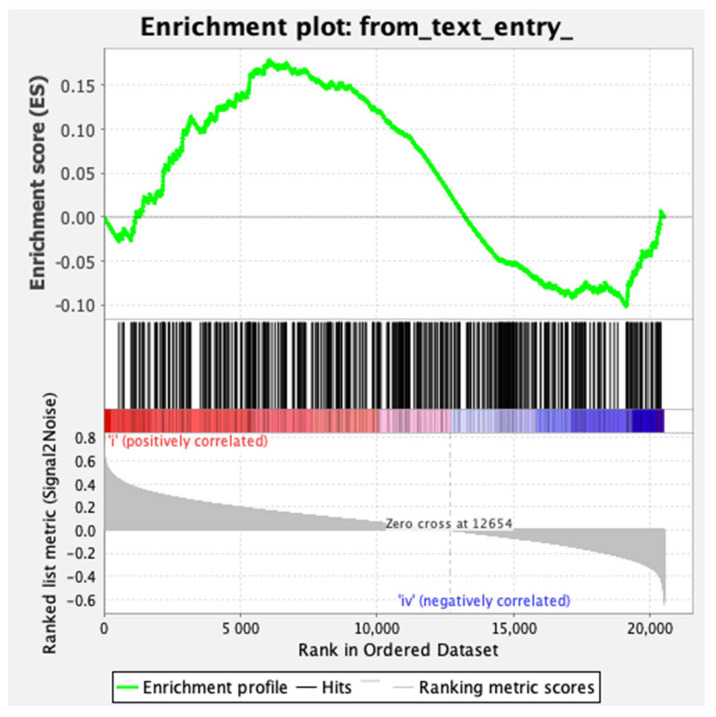
Gene set enrichment analysis (GSEA) of YAP/TAZ-regulated genes in relation to *CYLD* expression in breast cancer samples. Shown is the enrichment plot for the YAP/TAZ-regulated gene set correlated with reduced *CYLD* expression in stage I versus stage IV breast cancer cases. The enrichment score (ES) is indicated.

Given our observations in vitro and in vivo in mice, we next set out to investigate whether *CYLD* downregulation correlates with aberrant YAP/TAZ signaling in human breast cancers. For this, we used gene set enrichment analysis (GSEA) to determine how downregulation of *CYLD* expression associates with YAP/TAZ-regulated genes. A total of 996 stage- and *CYLD*-expression-stratified samples of human breast cancer cases from the Tumor Cancer Genome Atlas (TCGA) database were queried. Stage definition was based on the TNM system, which assesses the size of the tumor and whether it has grown into nearby tissue (T), whether cancer is present in the lymph nodes (N), and whether the cancer has spread to other parts of the body beyond the breast (M). The enrichment score was calculated for YAP/TAZ-regulated genes in stage I versus stage IV samples with reduced *CYLD* expression [46]. In total, 10% of the queried samples demonstrated underexpressed *CYLD*. As shown in Figure 7, a clear enrichment of the YAP/TAZ-regulated genes in *CYLD*-underexpressing samples was observed.

## 3. Discussion

The present report identified a novel mechanism of mammary epithelial cell identity maintenance, which depends on the coordinated regulation of YAP/TAZ and TGFβ pathways by the tumor suppressor CYLD. More specifically, our experiments demonstrated that either downregulation or carboxyl-terminal truncating mutations of CYLD are sufficient to induce mammary EMT. This was evident in two human mammary epithelial cell lines using two- and three-dimensional culture systems. In addition, the inactivation of CYLD in mouse mammary epithelia induced hyperplastic alterations, which are consistent with the changes that were observed in the cell line models. The mechanism that underlies this critical function of CYLD in mammary epithelial cells was analyzed further in the present study. Previous reports have identified a negative regulatory role for CYLD on the TGFβ pathway in lung and oral squamous epithelial cells [47,48,49]. However, the activation of the TGFβ pathway *per se* is known to have cytostatic effects in epithelial cells and this was confirmed in our analysis of wild type MCF10A cells [50]. Remarkably, the inactivation of CYLD circumvented the cytostatic effects of TGFβ pathway activation, while exploiting its EMT-inducing function to promote the attenuation of mammary epithelial cell characteristics. Our study demonstrated that this effect was achieved by the concomitant activation of the YAP/TAZ transcriptional regulators. Previous work from us had shown that the artificially induced activation of YAP/TAZ can offset the cytostatic activity of TGFβ in mammary epithelial cells and promote their tumorigenic properties [41]. The present study identified CYLD as the critical negative regulator of both TGFβ and YAP/TAZ activities in mammary epithelial cells that safeguards their epithelial identity. In fact, our study identified for the first time CYLD as a negative regulator of the YAP/TAZ transcriptional regulators in confluent culture conditions. Hyperactivation of the YAP/TAZ complex has previously been involved in the progression of breast cancer by promoting migration and invasion [51]. Our analysis indicates that one of the molecular alterations that can induce the activity of these transcription factors in mammary epithelial cells is the downregulation of CYLD. The molecular mechanism of CYLD-dependent regulation of YAP/TAZ is not clear at present. It is possible that CYLD affects the activity of YAP/TAZ by modulating the ubiquitination of these factors or specific upstream regulators. CYLD selectively hydrolyzes K63- or M1-linked polyubiquitin chains. Therefore, a possible mechanism of CYLD-mediated regulation of YAP/TAZ would involve the modulation of K63- or M1-linked protein polyubiquitination. Interestingly, K63-linked polyubiquitination of YAP promotes its nuclear localization and activity and the deubiquitinase OTUD1 was identified as the deubiquitinase that can reverse K63-linked polyubiquitination of YAP and promote its activation [52]. CYLD may act in a similar manner, yet the relevant targets of CYLD are not known at present. The identification of the proteins that are targeted by CYLD and regulate the YAP and TAZ factors will require a systematic analysis of protein ubiquitination in wild-type and CYLD-deficient mammary epithelial cells.

Our data identified CYLD as a key factor that coordinately regulates both the YAP/TAZ and the TGFβ signaling pathways to prevent mammary EMT. In agreement with previous studies, the coordinated activation of YAP/TAZ and TGFβ pathways conferred stem cell-like phenotypic characteristics in CYLD-deficient mammary cells and cancelled the growth inhibitory effect of TGFβ. These attributes are consistent with a phenotypic transition from a benign behavior to an invasive one, which can be triggered by the loss of CYLD protein function or expression, most likely at a late stage of cancer progression. It should be noted that EMT is not a binary process and several studies have identified intermediate stages in the transition from the epithelial to the mesenchymal state with potential implications for the progression of cancer [53,54]. At present, the detailed role of CYLD in the manifestation of the various stages of EMT is not clear. CYLD may affect a specific step in the EMT process or have a broader role in it. These questions will be addressed in future studies using temporally controlled systems of CYLD expression manipulation.

Despite the extensive evidence supporting a tumor-suppressing role of CYLD in breast cancer, one cannot exclude the possibility that CYLD may also play a tumor-promoting role in certain cases of breast cancer. This functional duality has been documented for other cancer-associated genes [55,56]. Interestingly, in one recently published study [57], analysis of tumor mRNA expression in a specific cohort of breast cancer patients from the TCGA database showed that high expression of *CYLD* was linked to shorter disease-free survival. The apparent contradiction between the study by Popeda et al. [57] and the study by Hayashi et al. [35] may be due to the fact that the former is based on mRNA expression data whereas the Hayashi study was based on protein expression data. Furthermore, it is possible that the role of CYLD in breast cancer development and evolution may depend on the particular type of breast cancer, as well as additional genetic and epigenetic alterations that exist in these tumors. Clearly, additional analyses of properly stratified clinical samples are needed to clarify these issues.

Overall, our study provides compelling evidence that could explain the association of CYLD downregulation at least with a subset of poor-prognosis invasive breast cancers and identify valuable elements of a growth regulatory framework that can be exploited for therapeutic and diagnostic purposes.

## 4. Materials and Methods

### 4.1. Cell Culture

MCF7 is a human breast cancer cell line that resembles the luminal type of breast cancer. MCF10A are immortalized human mammary epithelial cells. MCF7 and MCF10A cells were obtained originally from ATCC and they were authenticated by short-tandem repeat (STR) profiling (Eurofins Genomics Europe, Konstanz, Germany). MCF7 were grown in Dulbecco’s Modified Eagles medium containing 4.5 g/L of glucose (Gibco-Invitrogen, Waltham, MA, USA), 10% fetal bovine serum (Gibco-Invitrogen), and 100 U/µL of penicillin-streptomycin (Gibco-Invitrogen). MCF10A cells were cultured in (1:1) DMEM:F12 medium (Gibco-Invitrogen) supplemented with 5% horse serum (Gibco-Invitrogen), 100 ng/mL EGF (Peprotech, London, UK), 1 µg/mL hydrocortisone (Sigma-Aldrich, St. Louis, MO, USA), 10 µg/mL insulin (Cayman Chemical, Ann Arbor, MI, USA), and 100 U/µL of penicillin-streptomycin (Gibco-Invitrogen). The cells were maintained in an incubator at 5% CO_2_ with a controlled temperature of 37 °C.

### 4.2. Mouse Models

All animal experiments were approved by the Aristotle University of Thessaloniki Faculty of Veterinary Medicine Review Board for compliance to FELASA regulations and licensed by the National Veterinary Administration authorities (License No. 94,354/866). Mice (C57BL/6 and BALB/c) were kept in bio-containment facilities in individually ventilated cages, fed with sterilized regular mouse chow (Mucedola, Milan, Italy), and given sterilized water ad libitum. Genotyping of the mice was performed by polymerase chain reaction (PCR) analysis of genomic DNA. The following PCR primers were used to characterize the *CYLD* locus: FWD1: 5′-GATGGCTCTTGTCACCACTT-3′, Fn: 5′-GGATCACTGTTGCCATCCTT-3′, and Rn4: 5′-AAAAAGACCCCCAGCCTTTA-3′. The presence of the Cre transgene was assessed by PCR of genomic DNA using the following primers: 1084: 5′-GCGGTCT GGCAGTAAAAACTATC-3′, 1085: 5′-GTGAAACAGCAT TGCTGTCACTT-3′, 7338: 5′-CTAGGCCACAGAATTGAAAGATCT-3′, and 7339: 5′-GTAGGTGGA AATTCTAGCATCATCC-3′. The 1084 and 1085 primers amplify a 102-bp DNA fragment of the *Cre* transgene, whereas the 7338 and 7339 primers were used concurrently with the *Cre*-specific primers to amplify a 324-bp genomic DNA fragment as the internal control. All female mice of experimental groups were maintained on a mixed 1:1 C57BL/6:BALB/c background. Each experimental group included 8 female mice.

### 4.3. Generation of CYLD-Mutated MCF10A Cell Lines

For the stable generation of *CYLD*-mutated MCF10A cell lines, the lentiviral vector lentiCRISPRv2 [58,59] was used. Three different gRNAs targeting exons 2, 3, and 9 of the *CYLD* locus (http://www.ensembl.org/index.html, ENSG00000083799) and one control sgRNA targeting *GFP* were designed using the Crispr tool of the Benchling suite (www.benchling.com). The sgRNA-coding oligonucleotides were subcloned in the LentiCRISPRv2 vector using the BsmBI restriction sites. The sequences of the cloned oligonucleotides were verified by sequencing. All sgRNA sequences are listed in Table S1. Third-generation VSV-G pseudotyped high-titer lentiviruses were generated by transient co-transfection of HEK293FT cells without serum 2 h prior to transfection with a five-plasmid combination as follows: One 150-mm tissue culture dish containing 4.5 × 10^6^ cells was transfected using 10 mM PEI (Sigma-Aldrich) diluted with Opti-MEM (Gibco-Invitrogen) and mixed with 10 µg lentiviral vector, 3 µg pVSVG [60], 5 µg pADV [61], 4.15 µg pRRE, and 2.1 µg pREV [62]. Supernatants were collected every 24 and 48 h after transfection, pulled together, and frozen at −70 °C. For lentiviral transduction, 10^3^ MCF10A cells/well were seeded in 24-well tissue culture plates and infected the following day with all four different lentiviruses in the presence of 8 μg/mL Polybrene (Santa Cruz Biotechnology, Dallas, TX, USA). Two days post-transduction, cells were selected for 6 days with puromycin (250 µg/mL, Invivogen, San Diego, CA, USA), and monoclonal cell colonies were subcultured and established. Images were captured using an Inverted Primovert microscope (Zeiss, Oberkochen, Germany). The CYLD-deficient clones KO-B4, KO-C5, and KO-F2 were generated with gRNAs targeting exons 2, 3, and 9, respectively.

### 4.4. TGFβ Treatment and Cell Viability Assay

TGFβ (Peprotech) was reconstituted in citric acid (pH = 3.0) at a stock concentration of 100 μg/mL. Cells were seeded in 12-well cell culture plate (5 × 10^4^/well) and the following day were treated with 5 ng/mL TGFβ for 24 h. Cell viability was determined by the Trypan Blue exclusion assay (Sigma-Aldrich).

### 4.5. Small Interfering RNA (siRNA) Knockdown

First, 2 × 10^6^ cells were seeded in 6-well plates and allowed to grow overnight. The following day, after a dilution with nuclease-free water, cells were transfected with 10 nM of *CYLD*- [63], *SMAD2*-, *YAP*-, or *TAZ*-targeting siRNAs (Qiagen, Hilden, Germany) using 3 μL of Lipofectamine RNAiMax transfection agent (Invitrogen, Carlsbad, CA, USA) according to the manufacturer’s instructions. A control siRNA, targeting the luciferase gene [64], was used as a negative control. Images were captured using Inverted Primovert microscope (Zeiss).

### 4.6. Immunoblotting

Cells were rinsed twice with ice-cold phosphate-buffered solution (PBS) and lysed with SDS lysis buffer (50 mM Tris-HCl pH 6.8, 2% SDS, 10% glycerol, and 3% β-mercaptoethanol), followed by heating at 95 °C for 5 min. For the phosphorylated form of proteins, whole cell extracts were harvested in RIPA lysis buffer (50 mM Tris-HCl pH7.4, 150 mM NaCl, 2 mM EDTA, 1% NP-40, and 0.1% SDS) containing DTT 1 mM and protease/phosphatase inhibitor cocktail (Sigma Aldrich). The samples were analyzed by SDS-PAGE and proteins were electrophoretically transferred to nitrocellulose membrane for Western blot analysis. Blocking was done using 5% not-fat dry milk (Sigma-Aldrich) or 5% BSA (Sigma-Aldrich) for the detection of phosphorylated proteins, for 1 h at room temperature. Immunoblotting was performed using antibodies outlined in Table S2. Membrane-bound antibodies were detected by an enhanced chemiluminescence detection kit (Pierce, Waltham, MA, USA) using a Typhoon FLA 7000 imaging system (GE Healthcare Life Sciences, Chicago, IL, USA). Bands were quantified using ImageJ software (NIH, Bethesda, city, MD, USA).

### 4.7. RNA Extraction, cDNA Synthesis, and Quantitative Real-Time PCR (qPCR)

Nucleozol reagent (Macherey-Nagel, Düren, Germany) was used to extract total RNA from cells and 1 μg of total RNA was transcribed to cDNA using the RevertAid Reverse Transcriptase system (Fermentas, Waltham, MA, USA) and oligodT_18_. Analysis of cDNA samples by real-time qPCR was performed using the Applied Biosystems StepOne system and SYBR Green (Kapa Biosystems, Wilmington, MA, USA) according to the manufacturer’s instructions. The PCR program included 1 cycle at 95 °C for 10 min and 40 cycles at 95 °C for 15 s and at 60 °C for 1 min. The threshold cycle (C_T_) value for each gene was normalized to the C_T_ value for *YWHAZ*. Relative expression levels were determined by the ΔΔC_T_ method [65]. The sequences of primers used for qPCR are outlined in Table S3.

### 4.8. Flow Cytometric Analysis for CD44/CD24 Markers

MCF10A cells were collected using Trypsin (ThermoFisher Scientific, Waltham, MA, USA) and washed in PBS. First, 10^6^ cells were blocked with blocking buffer (PBS + 2% FBS) on ice for 10 min and incubated with anti-CD44-FITC (IM7, Biolegend, San Diego, CA, USA) and anti-CD24-PE (sc-19585 PE, Santa Cruz Biotechnology) conjugated antibodies in 1:200 and 1:100 dilution, respectively, for 30 min on ice. Cells were then washed with blocking buffer and CD44/CD24 markers were analyzed using a Partec CyFlow ML flow cytometer (Sysmex Partec, Görliz, Germany).

### 4.9. Three-Dimensional Mammosphere Formation

MCF10A cells were collected using Trypsin (ThermoFisher Scientific) and resuspended in Assay Media (same as MCF10A growth media, but with no EGF and 2% horse serum). At the same time, frozen Matrigel (Corning, New York, NY, USA) was spread evenly to each well of an 8-well chamber slide (Lab-Tek, Scotts Valley, CA, USA) and placed in a cell culture incubator for solidification. After counting the cells using a Neubauer hemocytometer (Marienfeld, Lauda-Königshofen, Germany), 5 × 10^3^ cells were resuspended in Assay Media containing 2% Matrigel (Corning) and 5% EGF (Peprotech) and were seeded to a well of a Matrigel-precoated 8-well chamber slide. Cells were allowed to grow in the incubator for 20 days, with fresh Assay Media containing 2% Matrigel and 5% EGF being added every 4 days. Images were captured every 5 days using an Inverted Primovert microscope (Zeiss).

### 4.10. Three-Dimensional Invasion Assay

A three-dimensional spheroid assay was performed to assess the invasive ability of MCF10A cells, as described before [66]. MCF10A cells were collected using Trypsin (ThermoFisher Scientific) and resuspended in MCF10A cell culture media. After counting the cells using a Neubauer hemocytometer (Marienfeld), a dilution was performed to allow for seeding of 10^3^ cells per 20 µL drop of cell culture media. Here, 40 drops for each cell line were seeded onto the lid of a 10 cm cell culture dish, while 5 mL of PBS were added to the bottom. Cells were incubated at 37 °C for 72 h to generate spheroids. After 72 h, spheroids were collected and combined with a 1:1 mixture of cold Matrigel (Corning) and collagen type I (Corning), and this viscous mixture was seeded into the center of the well on a 24-well plate. For each cell line, the material was enough for 3 independent 3-D cultures. The 3-D cultures were placed in the incubator for 30 min to allow polymerization of the mixture and 1 mL of cell culture media was submerged slowly to the 3-D cultures. For the monitoring of spheroid invasion, images were captured at 0 (after plating), 24, 48, and 72 h using an Inverted Primovert microscope (Zeiss). The invasive ability was quantified using the ImageJ software and expressed as the longest invasive distance originating from the spheroid, minus the radius of the spheroid. For every biological replicate, the invasive ability of one spheroid from each of 3 independent technical replicates was assessed.

### 4.11. Immunofluorescence

MCF10A cells and mammospheres were fixed with 4% paraformaldehyde (Santa Cruz Biotechnology) and permeabilized with 0.5% Triton X-100 (ThermoFisher Scientific) in PBS before incubating with 5% normal goat serum (ThermoFisher Scientific) to block non-specific binding. They were then probed with the primary and secondary antibodies outlined in Table S2. Nuclei were stained with DAPI solution (Biolegend). Immunofluorescent specimens were observed using a Zeiss LSM 780 confocal microscope or a Zeiss AxioObserver D1 microscope. For YAP/TAZ immunofluorescence, MCF10A cells were fixed with 4% paraformaldehyde (Santa Cruz Biotechnology) for 15 min, washed with PBS, and permeabilized with 1% Triton-X-100 (American Bioanalytical, Canton, MA, USA) for 15 min. After washing with PBS, cells were blocked with 2% BSA (Fisher Scientific, Waltham, MA, USA) in PBS for 1 h and then incubated with primary antibody (D24E4, Cell Signaling, Beverly, MA, USA) overnight at 4 °C. Cells were washed in TBST and secondary antibody (711-166-152, Jackson ImmunoResearch, Cambridge, UK) was added for 1 h at room temperature. Primary and secondary antibodies were diluted in 2% BSA in PBS. Nuclei were stained with Hoescht (Sigma-Aldrich). The intensity of the YAP/TAZ nuclear stain in the immunocytochemistry experiments was quantitated using CellProfiler as previously described [67,68]. Specifically, image channels for nuclei and YAP/TAZ were split, individual nuclei were identified as primary objects, and the integrated intensity of YAP/TAZ staining was measured in individual nuclei across multiple images from two repeated experiments. The average nuclear area for each condition was then determined, and the intensity of YAP/TAZ staining relative to the size of individual nuclei was calculated.

### 4.12. Immunohistochemistry (IHC) and Morphometry

Formalin-fixed mammary glands were embedded in paraffin, cut at 5 μm, and stained with immunohistochemistry (IHC). Sections were blocked with normal goat serum (dilution 1:20 v/v in PBS, Dako, Jena, Germany) at 37 °C for 1 h. Heat-induced antigen retrieval was performed with citrate buffer, pH = 6.0 for YAP/TAZ or with CC1 epitope retrieval solution (Ventana Medical Systems, Inc., Oro Valley, AZ, USA) for Ki-67. Primary antibodies for IHC included rabbit antibodies against YAP/TAZ (D24E4, Cell Signaling, dilution 1:100 v/v) and Ki-67 (ab16667, Abcam, Cambridge, UK, dilution 1:100 v/v). Rabbit primary antibody binding was detected with goat anti-rabbit polymer HRP (ZytoChem Plus, Berlin, Germany). Color was developed with diaminobenzidine substrate-chromogen (ThermoFisher Scientific/Lab Vision) and tissues were counterstained with hematoxylin.

The extent of hyperplasia was semi-quantitatively assessed in Ki-67-immunostained mammary gland sections from control and CYLD-deficient mice of a matched background (C57BL/6-BALB/c (1:1)). Each section was scored on the basis of the frequency of mammary gland profiles showing pseudostratified epithelia with ample Ki-67 positivity using a 0–4 scale. Grading was done according to the following scheme. No abnormal gland profiles (score 0); abnormal gland profiles <25% (score 1), 25–50% (score 2), 50–75% (score 3), and 75–100% (score 4) of the total gland profiles present in the section. To quantify proliferation in hyperplastic mammary epithelia, 5 × 40 high power images of mammary gland profiles were captured from each Ki-67-stained section, resulting in 40 images from each experimental group. Ten images per group were then randomly selected and Ki-67-positive and -negative nuclei of mammary epithelial cells were counted in each image. Proliferation data was recorded as the fraction of Ki-67-positive epithelial cell nuclei/total epithelial cell nuclei in each image. Cell counts were performed with the ImageJ image processing and analysis program. Results were statistically compared between groups using the Mann–Whitney U test. Images were captured using an Inverted Eclipse E500 microscope (Nikon, Tokyo, Japan).

### 4.13. Statistics

All datasets were taken from *n* ≥ 3 biological replicates, unless it is specified differently. Data are presented as mean ± SE. The calculation of *p* values was performed with an unpaired Student’s *t*-test with Excel (Microsoft Office) or one-way ANOVA, Tukey’s multiple comparisons test, and Mann–Whitney U test with GraphPad Prism software (GraphPad Software, La Jolla California USA);; *p* < 0.05 was considered significant.

### 4.14. Gene Set Enrichment Analysis (GSEA)

GSEA calculates the enrichment score (ES) by walking down the ranked-ordered list of genes, increasing a running-sum statistic when a gene is in the gene set and decreasing it when it is not [69,70]. The generated plot (Figure 7) contains in the middle a rank-ordered list of 500 YAP/TAZ-dependent genes (Table S4) identified as the most variable targets from a meta-analysis of a publicly available dataset of YAP-regulated mice gut epithelium organoids [46]. The top of this list (red color, Figure 7) contains genes upregulated in *CYLD*-downregulated samples. The bottom of the list (blue color, Figure 7) contains downregulated genes in *CYLD*-upregulated samples. Anytime a gene from the gene set is found along the list, a vertical black bar is plotted (hit). If most of the hits are at the top of the list, then this gene set is enriched in *CYLD*-downregulated cases. However, if they are distributed homogenously across the rank-ordered list of genes, then that gene set is not enriched in any of the gene expression profiles.

## 5. Conclusions

Our study identified CYLD as a critical regulator of EMT in mammary epithelial cells. CYLD prevents the acquisition of mesenchymal phenotypic characteristics by mammary epithelial cells through the coordinated suppression of TGFβ signaling and YAP/TAZ activation. Our findings explain the association of CYLD downregulation with aggressive breast cancers and lay the ground for the development of targeted therapeutic approaches of CYLD-deficient breast cancers.

## Supplementary Materials

The following are available online at https://www.mdpi.com/2072-6694/12/8/2047/s1, Figure S1: CYLD inactivation upregulates N-cadherin protein expression, Figure S2: *CYLD* downregulation leads to EMT-like phenotypic changes in MCF10A cells, Figure S3: *CYLD* downregulation induces EMT-like changes in breast cancer cell line MCF7, Figure S4: Quantification of the fluorescence intensity of vimentin and SMA staining of Figure 2, Figure S5: CYLD inactivation upregulates *CD44* and downregulates *CD24* mRNA expression, Figure S5: Evaluation of siRNA-mediated downregulation of CYLD, YAP and TAZ, Figure S6. Analysis of YAP, TAZ and CYLD expression by immunoblotting. Figure S7: Generation of mice with mammary-specific deletion of *CYLD* exon 9 (*MMTVCre-Cyld^fl9/fl9^*). Figure S8: Uncropped images of the Western blots shown in figures 1,4,S1, and S6. Table S1. sgRNA sequences used for CRISPR/Cas9-mediated *CYLD* mutagenesis, Table S2. Antibodies used in Western Blot and Immunofluorescence, Table S3. Primer sequences used in qPCR, Table S4. List of the top 500 genes with the highest variability between *YAP*-expressing and *YAP*-deficient mouse gut organoid cultures.
