# Supplementary material for: The Tumor Suppressor CYLD Inhibits Mammary Epithelial to Mesenchymal Transition by the Coordinated Inhibition of YAP/TAZ and TGFβ Signaling
Source: Cancers (Basel). 2020 Jul 24;12(8):2047. doi: 10.3390/cancers12082047 (PMC7466024; doi:10.3390/cancers12082047)
Supplement: Supplementary file 1 [file cancers-12-02047-s001.pdf]

Supplementary Materials: The Tumor Suppressor CYLD Inhibits Mammary Epithelial to Mesenchymal Transition by the Coordinated Inhibition of YAP/TAZ and TGFbeta Signaling

Athanasios Pseftogas, Konstantinos Xanthopoulos, Theofilos Poutahidis, Chrysanthi Ainali, Dimitra Dafou, Emmanuel Panteris, Joseph G. Kern, Xaralabos Varelas, Alexander Hardas, Christos Gonidas, Anastasia Tsingotjidou, Eudoxia Hatzivassiliou, and George Mosialos

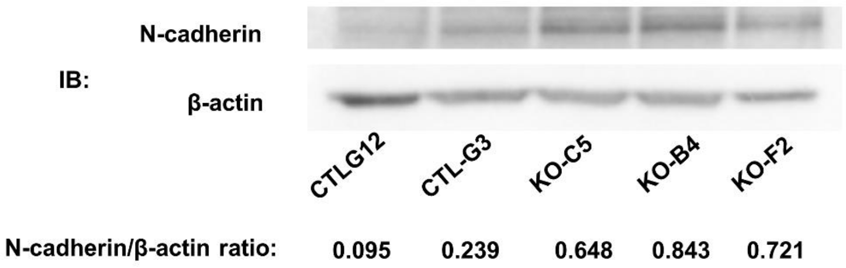

Figure S1. CYLD inactivation upregulates N-cadherin protein expression. Immunoblot analysis of N-cadherin and β-actin expression in whole cell extracts from control (CTL) and CYLD-deficient MCF10A cells (KO-B4, KO-C5 and KO-F2). The indicated ratios of band intensities are shown below the corresponding lane. Representative data from one out of two experiments are shown.

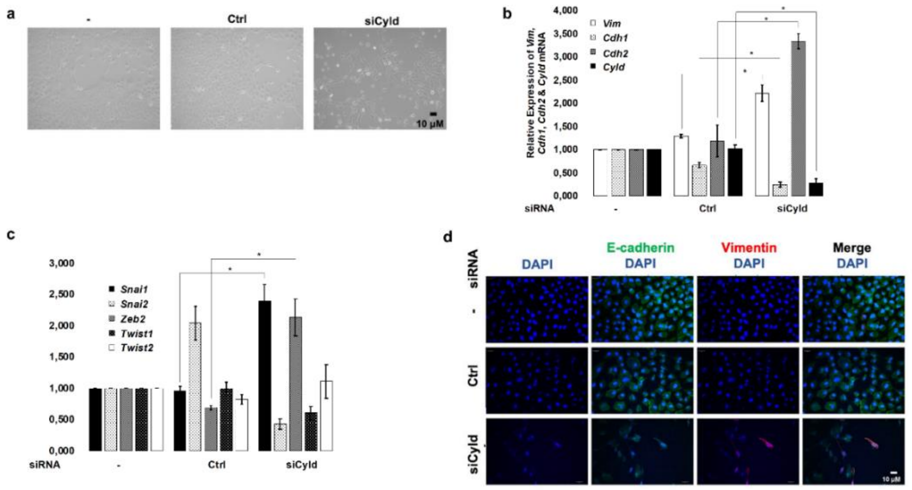

Figure S2. *CYLD* downregulation leads to EMT-like phenotypic changes in MCF10A cells. **a**) Morphological alteration of MCF10A cells with downregulated *CYLD* expression. MCF10A cells were transfected with luciferase-targeting (Ctrl) or *CYLD*-targeting (siCyld) siRNAs, and photographed 48 hours after transfection. **b**) *CYLD* downregulation leads to reduction of *E-cadherin* (Cdh1) and upregulation of *Vimentin* (Vim) and *N-cadherin* (Cdh2) mRNA expression levels. MCF10A cells were transfected with *CYLD*-targeting (siCyld) or luciferase-targeting (Ctrl) siRNAs. After 48 hours, total RNA was extracted and used to determine the relative levels of the indicated mRNAs using qPCR. The histogram indicates the average values (+/− SE) of relative mRNA levels as determined by the $\Delta\Delta C_T$ method and *YWHAZ* as endogenous control from at least three independent experiments. The statistical analysis of the pairwise comparisons indicated by brackets was performed by the Student's *t*-test method. (* $p \leq 0.05$). **c**) *CYLD* downregulation induces the mRNA expression of EMT-associated transcription factors Snaill and ZEB2. MCF10A cells were transfected with *CYLD*-targeting (siCyld)

or luciferase-targeting (Ctrl) siRNAs. After 48 hours, total RNA was extracted and used to determine the relative levels of the indicated mRNAs using qPCR. The histogram indicates the average values (+/− SE) of relative mRNA levels as determined by the ΔΔCT method using *YWHAZ* as the endogenous control from at least three independent experiments. The statistical analysis of the pairwise comparisons indicated by brackets was performed by the Student's t-test method. (* $p \leq 0.05$). **d**) Detection of E-cadherin and Vimentin by immunofluorescence in MCF10A cells treated as described in B. Green color represents the staining of E-cadherin, red represents the staining of Vimentin and blue represents the nuclear DNA staining by DAPI.

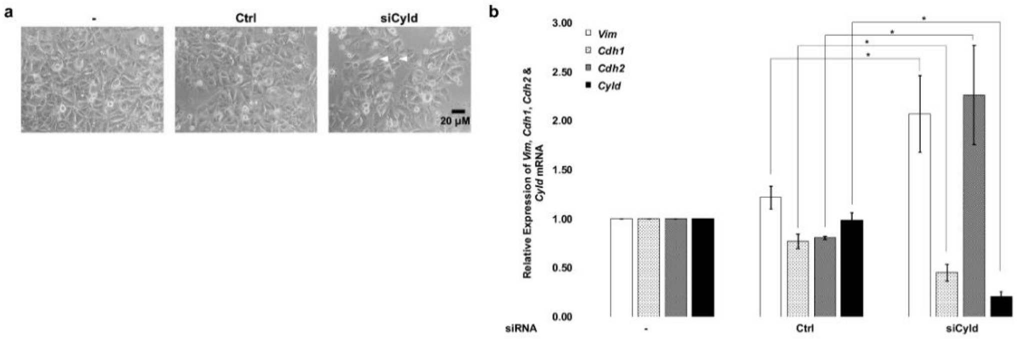

Figure S3. CYLD downregulation induces EMT-like changes in breast cancer cell line MCF7. **a**) Alteration of the epithelial phenotype of MCF7 cells to mesenchymal, following *CYLD* downregulation. MCF7 cells were transfected with luciferase-targeting (Ctrl) or *CYLD*-targeting (siCyld) siRNAs, and an EMT-like phenotype (white arrowheads) was observed in more cells with downregulated *CYLD* compared to controls, 72 hours after transfection. **b**) MCF7 cells were transfected with *CYLD*-targeting (siCyld) or luciferase-targeting (Ctrl) siRNAs. After 48 hours, total RNA was extracted and used to determine the relative levels of the indicated *Vimentin* (Vim), *E-cadherin* (Cdh1), *N-cadherin* (Cdh2) and *CYLD* (Cyld) mRNAs by qPCR. The histogram indicates the average values (+/− SE) obtained from at least three independent experiments using the ΔΔCT method and *YWHAZ* as endogenous control. The statistical analysis of the pairwise comparisons indicated by brackets was performed by the Student's *t*-test method. (* $p \leq 0.05$).

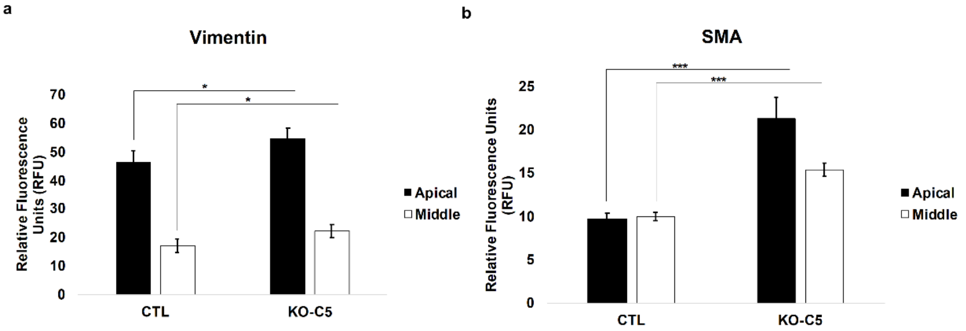

Figure S4. Quantification of the fluorescence intensity of vimentin and SMA staining of figure 2. The relative fluorescence intensity (RLU) of vimentin (**a**) and SMA (**b**) staining of apical and middle sections shown in figure 2 was determined by the ImageJ software. Average values of fluorescence intensities per unit area (+/− SE) from 12 random fields of each image are shown. The statistical analysis of the pairwise comparisons indicated by brackets was performed by the Student's *t*-test method. (* $p \leq 0.05$, *** $p \leq 0.001$).

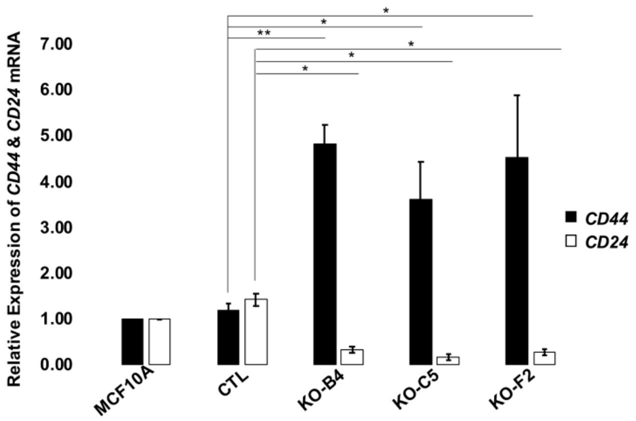

Figure S5. CYLD inactivation upregulates *CD44* and downregulates *CD24* mRNA expression**.** Total RNA was extracted from control (MCF10A, CTL) and CYLD-deficient MCF10A (KO-B4, KO-C5 and KO-F2) clones and used to determine the relative levels of the indicated mRNAs by qPCR. The histogram indicates the average values (+/− SE) of relative mRNA levels as determined by the ΔΔCT method and *YWHAZ* as endogenous control from at least three independent experiments. The statistical analysis of relative mRNA expression between the control clone and each clone of the CYLD-deficient clones was performed by the Student's *t*-test method. (* $p \leq 0.05$, ** $p \leq 0.01$).

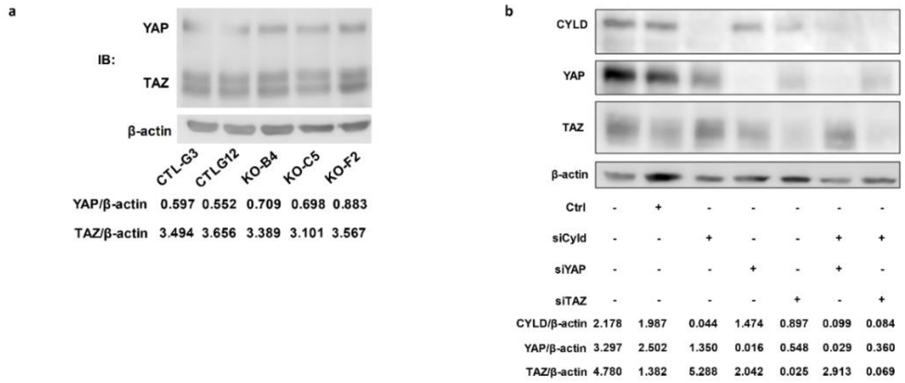

Figure S6. Analysis of YAP, TAZ and CYLD expression by immunoblotting. (**a**) Immunoblot analysis of YAP, TAZ and β-actin expression in whole cell extracts from control (CTL-G3, CTL-G12) and CYLD-deficient MCF10A cells (KO-B4, KO-C5 and KO-F2). The indicated ratios of band intensities are shown below the corresponding lane. (**b**)Evaluation of siRNA-mediated downregulation of CYLD, YAP and TAZ. Immunoblot analysis of whole cell extracts obtained from MCF10A cells that were transfected with *CYLD*-targeting (siCyld), *YAP*-targeting (siYap), *TAZ*-targeting (siTaz) or luciferase-targeting (Ctrl) siRNAs as indicated. The indicated ratios of band intensities are shown below the corresponding lane.

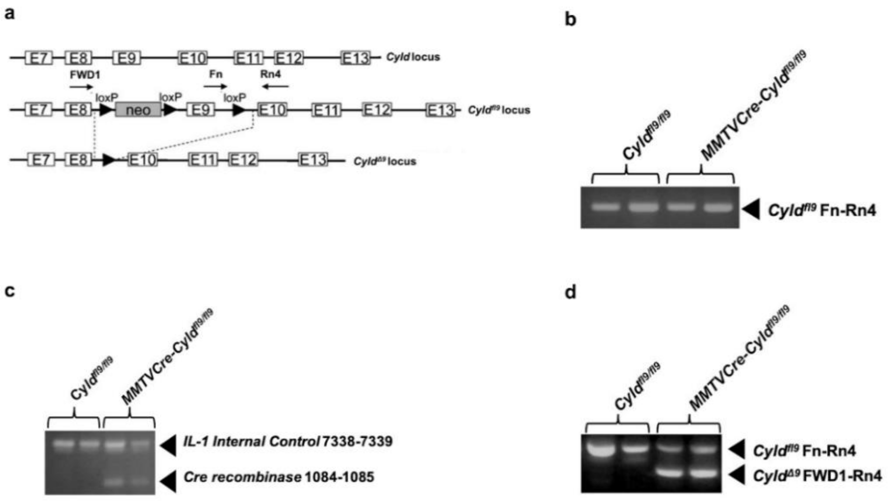

Figure S7. Generation of mice with mammary-specific deletion of *CYLD* exon 9 (*MMTVCre-Cyld*$^{fl9/fl9}$). **a**) Schematic representation of exons 7–13 (E7-E13) of the murine *CYLD* locus (*Cyld* locus). The structures of the floxed *CYLD* locus in the absence (*Cyld*^{fl9}locus) and presence (*Cyld*$^{\Delta 9}$locus) of the Cre recombinase are shown. The loxP sites are shown as solid triangles. The positions of primers FWD1, Fn and Rn4 are marked by arrows. **b**) PCR-based detection of the *Cyld*^{fl9} allele (*Cyld*^{fl9} Fn-Rn4) in genomic DNA obtained from the tails of *Cyld*$^{fl9/fl9}$ and *MMTVCre-Cyld*$^{fl9/fl9}$ mice. c) PCR-based detection of the *Cre* recombinase gene in genomic DNA obtained from the tails of *MMTVCre-Cyld*$^{fl9/fl9}$ mice. Genomic DNA from *Cyld*$^{fl9/fl9}$ mice was used as negative control. d) PCR-based detection of the Cre-mediated recombination product (*Cyld*$^{\Delta 9}$ FWD1-Rn4) in mammary gland genomic DNA isolated from *MMTVCre-Cyld*$^{fl9/fl9}$ mice. Mammary gland genomic DNA from *Cyld*$^{fl9/fl9}$ mice was used as negative control.

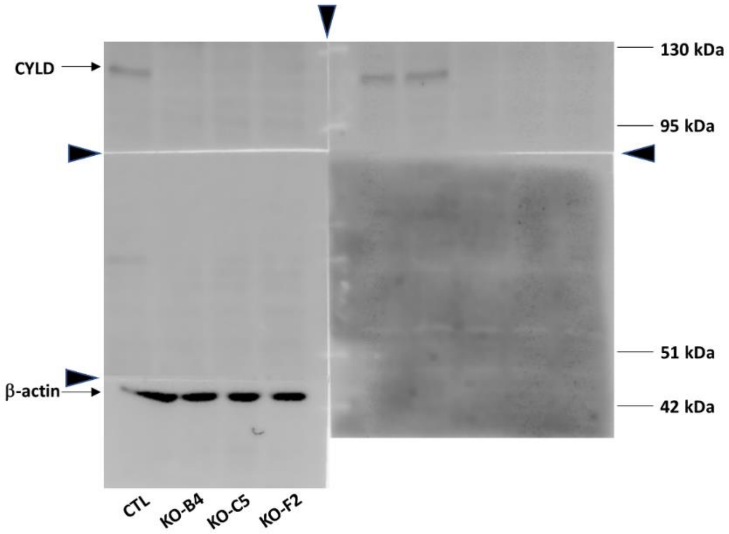

Uncropped image of Figure 1a.

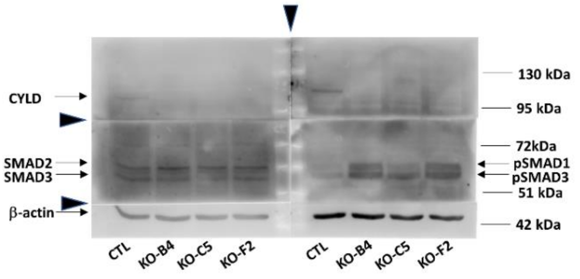

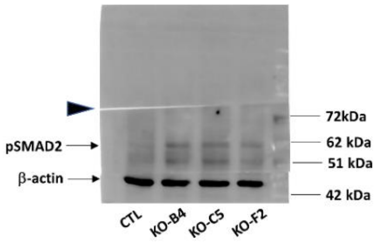

Uncropped images that were used for the composition of Figure 4a.

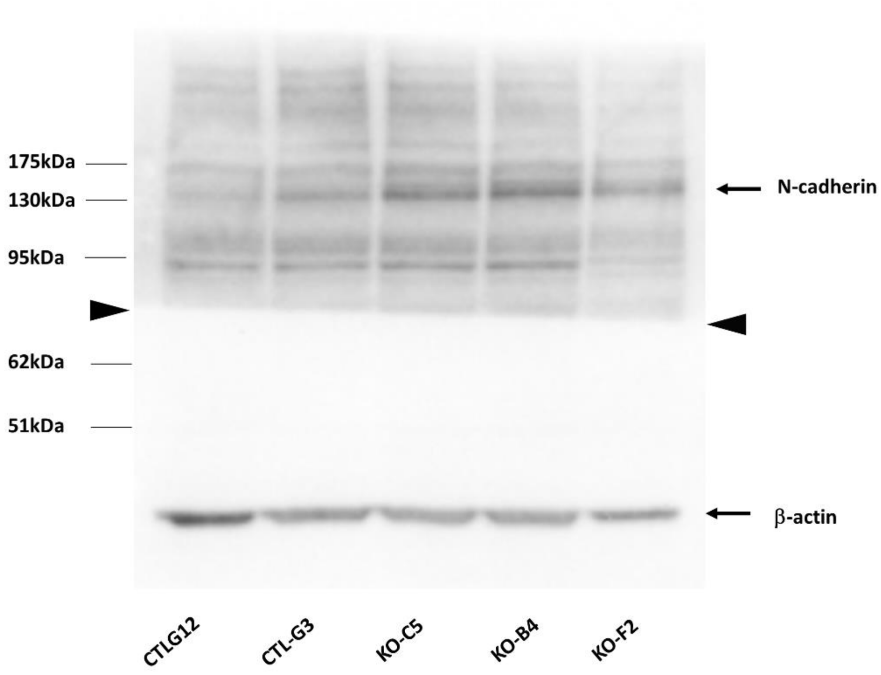

Uncropped images that were used for the composition of Figure S1.

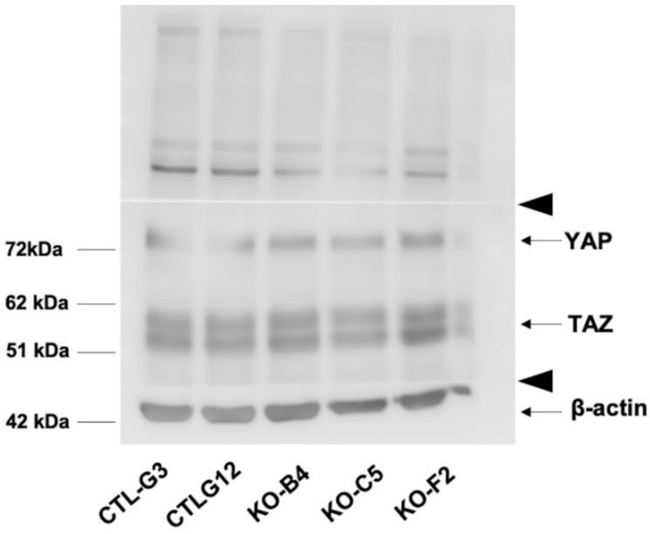

Uncropped image that was used for the composition of Figure S6a.

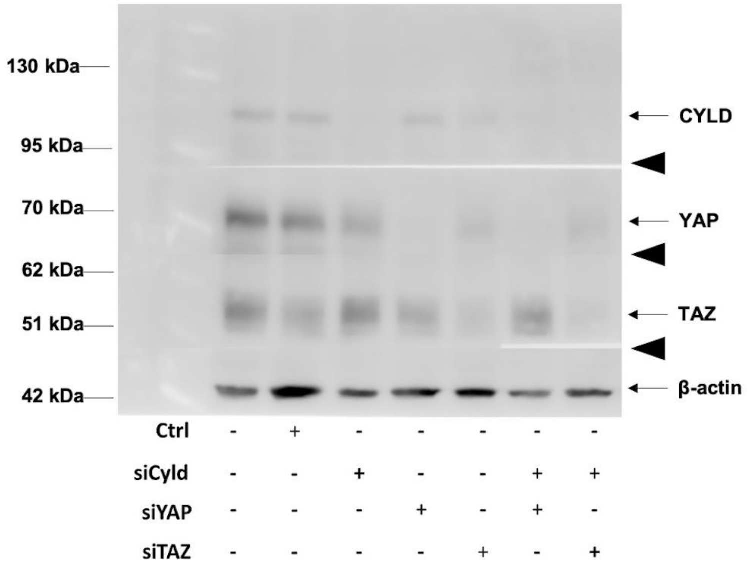

Uncropped image that was used for the composition of Figure S6b.

Figure S8. Uncropped images of the Western blots shown in figures 1,4,S1, and S6. The nitrocellulose membranes were cut at the positions indicated by the dark arrowheads and incubated separately with antibodies that recognize the indicated proteins. At the end of the immunoblotting process the membrane pieces were placed next to each other and scanned in order to obtain the provided images.

Table S1. sgRNA sequences used for CRISPR/Cas9-mediated *CYLD* mutagenesis.

Name	Start Site	Strand	Sequence (5′-3′)	PAM Sequence
2.3 (Exon 2)	50783781	+	ATCGTTCTGTGGGGCATTCA	AGG
3.2 (Exon 3)	50785678	+	GCATTGGAAAGTGATTACGC	AGG
9.2 (Exon 9)	50815200	+	GAGTGTGCAGGCTGTACGGA	TGG
Ctrl (GFP)			GTGGTGCAGATGAACT	

Table S2. Antibodies used in Western Blot and Immunofluorescence.

Specificity	Source	Catalog No.	Application (Concentration)
mouse anti-CYLD	Santa Cruz	sc-74434	WB (1:200)
mouse anti-β-actin	Santa Cruz	sc-47778	WB (1:1000)
rabbit anti-pSMAD3	Cell Signaling	9514	WB (1:1000)
rabbit anti-pSMAD2	Cell Signaling	3101	WB (1:1000)
goat anti-SMAD2/3	Santa Cruz	sc-6032	WB (1:1000)
rabbit anti-YAP/TAZ	Cell Signaling	8418	WB (1:1000)
rabbit anti-MST1	Upstate	07-061	WB (1:1000)
rabbit anti-MST2	Abcam	ab52641	WB (1:1000)
rabbit anti-pMST1/MST2	Genetex	GTX133948	WB (1:1000)
rabbit anti-N-cadherin	Cell Signaling Technology	13116	WB (1:500)
rabbit anti-E-cadherin	Santa Cruz	sc-7870	IF (1:50)
mouse anti-Vimentin	BioGenex	MU074	IF (1:50)
rabbit anti-Keratin-5	Biolegend	PRB-160P	IF (1:50)
mouse anti-α-SMA	Sigma Aldrich	A5228	IF (1:50)
donkey anti-mouse Alexa Fluor 555	ThermoFisher Scientific	A-31570	IF (1:200)
goat anti-rabbit Alexa Fluor 555	ThermoFisher Scientific	A-21430	IF (1:200)
donkey anti-mouse Alexa Fluor 488	ThermoFisher Scientific	A-21202	IF (1:200)
goat anti-rabbit Alexa Fluor 488	ThermoFisher Scientific	A-11070	IF (1:200)

Table S3. Primer sequences used in qPCR.

Gene	Forward (5′-3′)	Reverse (5′-3′)
Cyld	GATTCTGCCTGGCTCTTCTTT	CAGGTCTTCCAGAGACATCTTC
Cdh1	GAAAGCGGCTGATACTGACC	CGTACATGTCAGCCGCTTC
Cdh2	TGTTTGACTATGAAGGCAGTGG	TCAGTCATCACCTCCACCAT
Vim	TGTCCAAATCGATGTGGATGTTTC	TTGTACCATTCTTCTGCCTCCTG
Smad2	TCTGCTCGAGAAGCCAGAATGTGT	TCAGTCTGCATCAGGACACCCAAT
Ankrd1	AGTAGAGGAACTGGTCACTGG	TGGGCTAGAAGTGTCTTCAGAT
Ctgf	AGGAGTGGGTGTGTGACGA	CCAGGCAGTTGGCTCTAATC
Yap	AGAACTGCTTCGGCAGGC	TGGATTTTGAGTCCCACCAT
Taz	GTATCCCAGCCAAATCTCGTG	GGTTCTGCTGGCTCAGGGTACT
Snai1	CACTATGCCGCGCTCTTTC	GGTCGTAGGGCTGCTGGAA
Snai2	AAACTACAGCGAACTGGACACA	GCCCCAAAGATGAGGAGTATC
Twist1	AGTCCGCAGTCTTACGAGGA	GCCAGCTTGAGGGTCTGAAT
Twist2	CAAGCTGAGCAAGATCCAGAC	GGTCATCTTATTGTCCATCTCG
Zeb2	CGGTGCAAGAGGCGCAAACA	GGAGGACTCATGGTTGGGCA
Ywhaz	GCTGGTGATGACCAAGAAAGG	GGATGTGTTGGTTGCATTTCCT
Cd24	TGAAGAACATGTGAGAGGTTTGAC	GAAAACTGAATCTCCATTCCACAA
Cd44	GGAGCAGCACTTCAGGAGGTTAC	GGAATGTGTCTTGGTCTCTGGTAGC
Grhl2	CTCAGTATGACGTGCCCTCGCTG	GGTGGCTTCCAGGGTGTACTGAA
Ovol2	GCCCAAAGTCTTCCTGGTGAA	TAGGCCCACTGGGATGTAGGT

Table S4. List of the top 500 genes with the highest variability between *YAP*-expressing and *YAP*-deficient mouse gut organoid cultures. We have utilized differentially expressed genes from RNA sequencing data analyses previously deposited in the GEO repository under accession number GSE66567. RNA sequencing experiments were performed with RNA isolated from gut organoid cultures derived from at least three independent YAP deficient mice with genotypes *Yap*$^{fl/+}$*;villin-cre* (Day1_Het), *Yap*$^{fl/fl}$*;villin-cre* (Day1_KO) and *YapTg* (Dox_minus_Yap and Dox_plus_Yap). Variability across genes within samples facilitated the detection of genes that are driving YAP-dependent regeneration and tumorigenesis. We estimated the variance for each row in the logcounts matrix (`var_genes <− apply(logcounts, 1, var) and selected the top 500 genes with the highest variability and defined this data-set as YAP-dependent for future queries.

Number	GENE	Day1_Het	Day1_KO	Dox_minus_Yap	Dox_plus_Yap
1.	Yap1	6.083647053	4.191337689	6.60851978	11.99721424
2.	Ly6a	4.973621891	3.819204511	5.752808394	10.45752701
3.	Msln	4.695308415	3.027174135	4.739229516	9.528167856
4.	Ly6c1	2.951844214	2.872264567	3.045506992	8.159939056
5.	Serpinb9b	3.012299269	2.855188039	2.967615615	8.031139508
6.	Psca	3.338859537	2.937903966	3.183400944	8.222447696
7.	Bcmo1	7.520959489	8.713672167	3.973807683	3.769045196
8.	P2rx2	3.064229507	3.003281953	3.087635152	8.030037536
9.	Fabp1	13.61612908	14.2178359	12.7715964	8.728274331
10.	Cyr61	3.345061999	3.228191357	3.583969739	8.299661011
11.	Reg3a	9.351332175	9.90324439	8.633866971	4.51463999
12.	Isx	5.513251929	3.10795093	8.063333752	8.050353512
13.	Dusp14	3.599761904	3.458549252	3.747726488	8.344850209
14.	Lor	3.117513532	3.039643155	3.035650089	7.781450908
15.	Mt2	12.33662637	12.68985383	11.19582087	7.61186832
16.	Amotl2	3.270466766	3.073145912	3.325480668	7.790975565
17.	Anxa3	6.254805471	4.796730169	6.151644851	10.08103714
18.	Jub	4.026440167	3.203869608	3.998075386	8.185741598
19.	Gm16721	7.388784414	6.790325	7.490096701	11.67025911
20.	Clca6	6.063414383	5.765476732	6.187147309	10.47332736
21.	Cyp2d26	9.231669335	9.552318613	8.289714272	4.731232722
22.	Reg1	12.01118396	11.362241	10.97051169	7.122542131
23.	Epha2	4.943961884	3.861247998	5.026234935	8.773417809
24.	Gm9000	9.709317789	9.091039209	5.660160488	5.75358444

25.	Tinagl1	4.184559044	3.965078261	4.415596814	8.457457852
26.	Defa21	7.710738014	7.912004785	11.61108934	11.20302506
27.	Krt7	6.822275253	5.770599607	6.916377	10.52046805
28.	Ces1f	7.646381625	7.84001707	6.685406291	3.527039031
29.	Gprc5a	5.623480403	4.349881282	5.796267641	9.021139211
30.	Ahnak	4.56837767	3.844383685	4.628563616	8.245241781
31.	Plaur	5.500034979	3.746979625	5.059581508	8.427538692
32.	Olfm4	9.801222555	10.7001406	10.58319189	6.546582614
33.	Gm17367	8.622263304	9.226676955	7.889619281	4.855682569
34.	Gstm2.ps1	12.30325942	12.33977164	11.40298601	8.232427579
35.	Akr1c14	6.993996421	7.712793444	5.71882556	3.308834606
36.	Capn2	5.260712422	4.649340051	5.221728628	8.871300179
37.	Gstm1	11.17295755	11.23561964	10.21101498	7.13789546
38.	Gm17269	9.40186491	9.631877102	8.365912946	5.465234343
39.	Pmp22	4.375062394	3.682887574	4.530987548	7.925027403
40.	X1700019G06Rik	6.014657267	5.651795657	6.852986603	9.827292278
41.	Ctgf	3.098161828	2.903090125	2.960849596	6.772292558
42.	Leap2	7.581510969	7.802746759	6.932626555	3.733386678
43.	Cyp3a25	7.372869035	7.614826359	6.945646287	3.587008661
44.	Edn1	3.876483127	3.043796726	3.580449455	7.192670697
45.	Ano1	3.825245774	3.215881387	4.019703432	7.351344023
46.	Emp1	7.364036682	6.759475893	7.538611078	10.88505211
47.	Gm8074	7.645560594	7.342107213	4.931830206	3.816023652
48.	Ugt2b5	8.549374293	8.599221314	7.497439023	4.635383158
49.	Plk2	4.387418653	4.449425167	4.13728511	8.016167349
50.	Bmp2	3.881626726	3.462160329	3.886267407	7.413629818
51.	Lamc2	5.110928461	4.006036473	5.35609331	8.303635479
52.	Adh1	11.69026092	11.65742825	10.59051016	7.785682692
53.	Phldb2	3.575438857	3.219552205	3.84855995	7.141999541
54.	Defa23	5.215290094	5.307774088	8.577103734	8.210651994
55.	Krt80	2.979976223	2.871784374	2.9780279	6.571707902
56.	Slc5a4b	7.399116606	7.870634283	6.439884041	3.817744119
57.	Fabp6	5.731564482	4.714290576	8.320470761	4.286820034
58.	X1810030J14Rik	6.923762019	7.274616027	4.527787798	3.574028851
59.	Isg15	4.128985459	4.147638529	3.83941797	7.640632772
60.	Cyp3a11	8.637691277	8.691226611	7.261405374	4.846034467
61.	Ggt1	8.246503079	8.413907974	7.288113205	4.541533459
62.	Vnn1	4.082804677	3.55200068	3.727742329	7.322990398
63.	Il33	3.233285895	2.886126617	3.221654145	6.657438268
64.	S100a14	6.469037639	5.310458656	6.367060127	9.43359858
65.	Rgn	7.498004049	7.607501802	6.165791253	3.813442299
66.	Myo1c	5.527010556	4.997056691	5.605851936	8.857231619
67.	Wwc2	3.055976052	2.903370635	3.119131986	6.531614888
68.	Ier3	4.995198346	4.543365868	4.364502482	8.096130568
69.	Clic3	3.234402907	3.004585431	3.109805456	6.610607666
70.	Arg2	9.46718182	9.657656547	8.998752371	5.919671566
71.	Gcnt1	3.157502179	3.149104872	3.098681809	6.606980948
72.	Gstm3	11.13994865	11.13049787	10.66347289	7.536326123
73.	Myof	3.578253127	3.351870617	3.859293084	7.021497079
74.	X2210407C18Rik	10.51668423	9.360002535	8.938888205	6.427548024
75.	Fbp1	7.442974062	8.170840659	6.781241058	4.211169773
76.	Cyp2c68	7.759091113	7.933863813	6.33895531	4.218767393
77.	Sprr2a2	7.463213241	4.765595546	5.990544402	8.688207827
78.	Rdh7	7.599130643	7.941351008	6.498447682	4.157783768
79.	Ces2a	10.41664963	10.37773812	9.767596348	6.828259645
80.	Defa22	8.763883273	9.108147351	12.06713852	11.65217126
81.	Prss23	4.756585647	4.459117906	5.344226934	8.159513253
82.	Atp11a	3.533689824	3.18037675	3.577671557	6.783499006
83.	Treh	7.366083593	7.801766472	6.541252552	4.03169283
84.	Clu	4.060378423	3.17348463	4.065937452	7.01573677
85.	Sprr2a3	10.97382541	8.257643964	9.582432798	12.10597388
86.	Fgd3	3.142216131	2.904963117	3.076119715	6.369711313
87.	Bex1	3.140610639	3.07723685	4.082780622	6.613366978

88.	Wfdc2	3.247829073	2.940486509	3.50554192	6.496012965
89.	Syt8	3.879107819	2.884848455	3.405173618	6.540348069
90.	Cyp2c29	10.22064674	10.18981435	8.280032653	6.904553801
91.	Akr1b7	6.960405857	7.097927916	5.906677955	3.625259269
92.	Ugt2b36	7.333983406	7.139780727	5.969029111	3.840888659
93.	G6pc	6.127008569	7.165436269	6.085603684	3.435203605
94.	Lgals3	8.223649277	6.816367069	7.486101953	10.47504547
95.	Unc13d	3.373529355	3.002044385	3.339025445	6.396240062
96.	Rnf39	3.570517771	3.479383358	3.608616024	6.722023741
97.	Flna	4.095639168	3.665791921	4.411803249	7.119208007
98.	Ptrf	3.548293427	3.186187337	3.489232698	6.512058686
99.	Hsd17b13	7.899124902	7.676827324	5.988823109	4.58927883
100.	Ces1d	6.278880941	6.416778076	5.258825666	3.054432983
101.	Tnfrsf12a	6.867832053	5.928442994	6.70033099	9.494010465
102.	Slc13a1	7.155169118	7.090547282	6.782431025	3.925746889
103.	Ppp1r2	5.367606673	5.103388548	5.173637391	8.304701734
104.	Rpl15.ps2	6.94629549	6.091760197	3.817065556	4.008546312
105.	Lama5	3.141099544	3.063670983	3.294019142	6.227916129
106.	Apoa4	11.54668295	11.57294937	10.32274567	8.321544415
107.	Akr1c19	9.82269883	9.644475281	8.900705982	6.511923361
108.	Fabp2	11.90406469	12.29312444	10.76060167	8.899194791
109.	Mme	8.005225112	8.347281252	7.551043728	5.014357928
110.	Gm15308	8.12113246	8.655010662	11.1956315	10.68043791
111.	Zfp37	3.248399597	3.048978614	3.357209863	6.198477368
112.	S100g	11.15546524	10.79928418	9.868274292	7.83530275
113.	Dnase1	6.514247171	7.092070322	5.782711322	3.692459222
114.	Gsta3	7.755230363	7.81476674	5.678911201	4.89439375
115.	Wwc1	6.423270206	5.426816997	6.579910222	8.888238441
116.	Bco2	7.123150229	7.320103102	6.155502956	4.123750583
117.	Mt1	12.52415775	12.88452899	11.5433719	9.624930794
118.	Slc25a45	7.484133296	7.587676965	7.045078203	4.492488156
119.	Atf3	5.046445532	4.150103793	4.845720068	7.482726687
120.	Cnn2	4.649143394	4.129123919	4.193144653	7.137192119
121.	Cyp1a1	5.897297523	2.886013969	3.022329036	3.295241622
122.	Adam8	3.153332491	2.950821861	3.090912927	5.90817745
123.	Defa20	8.632480887	9.059632189	11.49037875	11.04704042
124.	Cybrd1	7.324727303	5.18220909	6.129067553	3.978205932
125.	Ctrb1	5.624503167	6.087014232	4.100407895	2.994753562
126.	Gm17532	3.04767185	2.849195706	3.09471474	5.827592539
127.	Slc2a5	7.061734722	7.774893132	6.920446246	4.518321211
128.	Gstm6	7.634438421	7.678705178	6.876034494	4.660298364
129.	Adh4	7.583622852	7.127175254	6.887463311	4.435665822
130.	Ehd2	2.982978885	2.873879104	2.961235776	5.745208413
131.	A430105I19Rik	3.260185407	3.215427886	3.21784956	6.032834685
132.	Anxa1	4.605598125	4.04787906	3.966527624	6.945367062
133.	Gstm4	7.978777195	7.895194331	6.975071268	4.97374551
134.	Khk	9.619297607	10.0626205	8.892145171	6.902411848
135.	Omt2b	6.005166589	5.720745202	3.810291245	3.189950327
136.	Nbl1	4.680836636	3.568172523	4.903604962	6.904929084
137.	Slc5a6	4.134179731	4.056790963	4.446064113	6.955796333
138.	Rbp2	12.3156931	12.42642686	11.66214676	9.459891782
139.	Thbs1	3.195189105	3.220115694	3.256553626	5.972593455
140.	Pard6b	5.915834123	5.14249677	5.891954274	8.291099386
141.	Abp1	8.145277433	8.390421205	8.180893244	5.520490143
142.	Asah2	7.135271446	7.347491362	5.820027551	4.415598019
143.	Spink3	8.152212261	8.842261216	7.822478193	5.7033068
144.	Mboat1	6.05674516	5.269657972	6.166088602	8.41523122
145.	Epn3	4.746107445	3.704375213	4.49379299	6.868923391
146.	Rras2	6.446175475	6.14577173	6.398842041	9.005860439
147.	Gm10972	4.945949881	4.354435835	4.096647101	7.039702872
148.	Cox7a1	8.038775392	8.591255156	7.256102296	5.530093323
149.	Lct	6.977276793	7.432701736	5.932149587	4.430992125
150.	Tigit	2.849195706	2.869338544	2.961444517	5.547578385

151.	Sord	7.353669991	7.924271752	6.519068787	4.876540397
152.	Lamb3	5.422243739	4.336546717	5.037399921	7.411490654
153.	Flnb	6.520069359	6.173076553	6.90438953	9.092302755
154.	Slc5a5	2.887913133	2.939695272	2.995128025	5.55986243
155.	Cdkn1c	4.204273236	4.455126434	3.725640121	6.676243883
156.	Clic4	5.1085224	4.770424729	5.202229227	7.590743279
157.	Ces2e	9.793164376	10.01609365	9.328895207	7.189050181
158.	Akr1c13	9.889426616	9.748614777	9.04565695	7.086043498
159.	Abcc5	3.658811868	3.328791348	3.736861019	6.129596711
160.	Hsd17b6	5.62633701	6.022278222	5.340275845	3.146626257
161.	Aldob	13.54051211	14.01779013	13.12262849	11.09290509
162.	Oasl2	3.760024903	3.856376665	3.766906607	6.358643721
163.	Klf6	6.429868541	5.843866013	6.237866003	8.672139884
164.	Tjp1	5.967569357	5.782794006	6.380199155	8.531004725
165.	Rin3	3.355869876	3.201208165	3.419958286	5.840483727
166.	Cd55	4.283805413	3.811622747	4.37681573	6.629062976
167.	Ifit1	3.456192626	3.30829741	3.456933869	5.921235837
168.	Ddx60	3.056447021	3.012874523	3.215536526	5.602940609
169.	S100a11	8.694410585	7.67148175	8.518931994	10.64423972
170.	Ephx1	8.64012257	8.735163465	7.635696922	6.03268188
171.	Apol9a	3.042260877	3.134998377	3.034285786	5.577297915
172.	Gm7861	9.312715618	9.75214604	11.85772196	11.45891899
173.	Ces1e	5.997000068	6.000313971	5.340590595	3.357914069
174.	Abhd2	6.645941671	6.090432303	6.391315433	8.830622016
175.	S100a6	10.51604688	9.354213257	10.32555035	12.34354374
176.	Chrnb1	3.427389182	3.141600823	3.625269461	5.859482145
177.	Sorbs2	3.236005649	3.127289701	3.453564386	5.746968401
178.	Slc26a3	6.70767879	6.71153908	6.447552275	4.147335086
179.	Fosl1	3.657938623	3.251087953	3.533064952	5.942046617
180.	Apol9b	3.008285107	3.101512233	3.037803165	5.528946963
181.	Kifc3	4.493668935	3.870978466	4.258315864	6.634787115
182.	Ccdc68	4.654557295	4.43302989	4.537003763	7.013231522
183.	Gm17664	3.23930883	3.812781934	4.31258294	6.099249233
184.	Rec8	5.715568189	6.195697221	4.8599315	3.383348153
185.	Gjb3	4.330201803	4.167922566	4.683019969	6.823261359
186.	Krt18	10.19643452	9.760660922	9.888429115	12.37724275
187.	Sema3c	3.848428412	3.656543152	3.912512573	6.248526009
188.	Tuft1	4.912585407	4.469392566	4.870832463	7.162500809
189.	Acot1	6.51732592	6.698607739	5.302491779	4.071875175
190.	Slc5a1	9.95067673	10.29300221	9.706989702	7.59515171
191.	Tmem86a	7.157351374	7.328645318	6.841048742	4.708674844
192.	Rbp7	5.880057941	6.151223435	5.8754437	3.5500462
193.	Pdlim2	3.941592387	3.422026993	4.000277803	6.1641167
194.	Neu1	5.395310998	5.042356606	5.88771198	7.761835762
195.	Slc5a11	5.471002484	5.862567912	5.079391586	3.143002504
196.	Hdac7	3.260234091	3.078921793	3.404028056	5.644947763
197.	Nt5c1a	3.043800057	2.849195706	3.274781788	5.439835736
198.	Tead4	3.155154053	3.034973215	3.251835286	5.549842113
199.	Lhfpl2	3.526168815	3.065762909	3.706006204	5.778841923
200.	Pcsk6	4.235400572	3.410756946	4.159696796	6.224157657
201.	Afp	7.28101574	7.216632748	6.139510727	4.71409952
202.	Pgap1	5.49141384	4.855761305	6.161401534	7.657856986
203.	Gm10020	6.712568584	6.074927266	4.041427663	4.841872967
204.	Cxcl16	5.649691605	4.350423078	5.32944733	7.242082051
205.	Lmo7	6.293782524	5.831606783	6.25235121	8.487048146
206.	Pigr	10.5184796	10.68250311	10.67853435	8.240817991
207.	Ppl	4.527562658	4.129576295	4.682784498	6.789938426
208.	Phlda1	6.816765827	6.251662322	6.875269636	8.969856608
209.	Apoc3	7.017731045	7.011987521	6.121401392	4.494754635
210.	Gm7665	8.00026318	7.420341276	7.879065526	10.08069999
211.	Gm17590	4.49551905	4.524526717	5.396179673	7.016554619
212.	Slc6a19	7.704647929	7.701257387	7.654064847	5.326707553
213.	Cwc22	4.044960176	3.864358437	5.95521568	6.031538103

214.	Mogat2	8.542176786	8.769746543	8.048038536	6.175076205
215.	Gm17379	6.179730345	7.206998002	4.362783494	5.892411082
216.	Cidec	5.386739959	4.719227879	5.004457764	7.31781101
217.	Lrrfip1	5.160861626	4.751384601	5.353276035	7.377553923
218.	Mgst1	9.065402167	9.44998489	8.431553319	6.804147024
219.	Apoc2	8.862358261	9.103833311	8.151420227	6.51643401
220.	Klf4	5.39040256	4.982472237	5.156760558	7.485508613
221.	Lars2	10.95389212	9.051272536	8.65859138	8.375309026
222.	Acaa1b	5.485733777	6.055429453	4.645622933	3.381966227
223.	Hbegf	4.252207339	3.723429442	4.359223834	6.356214433
224.	X2310007B03Rik	3.720729435	3.283909148	3.607069262	5.818830948
225.	Maob	7.796084134	7.848650328	7.30632887	5.39215698
226.	Ppp1r15a	4.487085669	4.052292589	4.297159887	6.556525983
227.	X2010106E10Rik	8.630078457	8.63842054	7.996501405	6.197559747
228.	Camk1	3.168763696	3.181331849	3.419038671	5.547823612
229.	Fam129a	3.617997764	3.100018417	3.750122065	5.722719628
230.	Syde1	3.000203876	2.861028797	3.078974008	5.274287094
231.	Gngt2	3.551436736	3.034498619	3.60443669	5.639892673
232.	Aldoc	6.421682062	6.228938728	5.280494024	3.904968153
233.	Nat8	7.074637302	7.121679106	6.1610901	4.665557605
234.	Agr2	9.151296831	9.262344884	9.577939193	7.062535398
235.	Hspa1b	4.293273841	3.477970222	3.916111625	6.090751267
236.	Vwce	5.601289751	6.040945036	5.554411627	3.489437686
237.	Cda	8.894256268	8.978612188	7.807655749	6.540522221
238.	Slc10a2	4.820988364	4.275333035	5.832560424	6.854206628
239.	Gm17438	5.383577205	5.858526014	6.762663411	7.966113062
240.	Dusp1	3.739126578	3.234611578	3.503343081	5.724619984
241.	Id2	5.503527816	5.380645877	4.904143922	7.470145125
242.	Slc35e4	3.561552564	3.283673021	3.517059791	5.705776779
243.	Ces1g	5.277822633	4.951584029	3.524238993	2.909194219
244.	Ndrg1	8.219126802	7.731759769	7.334050936	5.61652262
245.	Cyp2c55	7.958753252	7.640617601	6.445853626	5.505569462
246.	Adh6b	5.444264048	5.828846294	4.824270669	3.266793771
247.	X1810046K07Rik	5.387056332	5.518776924	4.510053493	3.069363917
248.	Arhgap40	3.396458548	3.043578848	3.322754782	5.484740712
249.	Aldh1a7	6.719131437	6.674567857	5.466154115	4.358956326
250.	Capn5	5.210006633	4.469092423	5.155791937	7.085485118
251.	Ccl25	8.907200364	9.085341077	8.686565626	6.677533562
252.	Klk1	6.983152259	7.487446995	6.144700638	4.928058263
253.	F3	6.20808728	4.810499813	5.813717659	7.508568513
254.	Ildr1	3.678286695	3.448800371	3.463825766	5.74757391
255.	Pbld2	7.084180127	7.212220303	6.664219318	4.810590923
256.	Pm20d1	6.063844208	5.86313808	4.958652943	3.625371868
257.	Id1	5.569855343	5.202119806	4.979378344	7.412441617
258.	Hmox1	4.191521848	3.358048795	4.152546324	5.978929981
259.	Ankrd37	6.376915324	5.543370899	5.330946484	3.730092309
260.	Tmigd1	4.301657881	4.104641054	5.705537592	6.395903459
261.	Mt4	5.028898901	5.190846619	3.648431357	2.901681068
262.	X1190002H23Rik	5.85753169	6.116877815	5.686066852	3.710871526
263.	Hpgds	5.577944199	6.399301935	5.275922558	3.761986734
264.	Serpinb9	3.050111035	2.962065897	3.093809849	5.234596577
265.	Bnip3	6.122625486	5.355997262	4.646983037	3.53410649
266.	Hmg1l1	3.32100504	3.24654749	5.093002582	5.266728171
267.	Ccl9	5.112592888	5.661701575	4.892484353	3.126237576
268.	Aldh1a1	10.86617247	10.94486051	9.957044685	8.59442716
269.	Slc25a48	3.424946832	3.176678072	3.768158987	5.588666633
270.	Dpep1	7.957108194	8.218066352	7.450629694	5.786208932
271.	Dak	10.25263545	10.71987845	9.807153533	8.207668026
272.	Pdcd4	6.712553518	6.881930936	6.618289996	4.567165907
273.	Jdp2	3.039522516	2.930257032	3.260010568	5.235924534
274.	Ldhd	4.070105149	3.491312271	4.168110159	6.00007899
275.	Anln	4.751900758	4.70020593	5.357059745	7.022742858
276.	Ociad2	7.467853775	7.611705943	7.105767627	5.270445987

277.	Defa25	6.846982044	7.563807876	9.171966615	8.814789543
278.	Slc6a20a	7.804911225	7.779009283	7.773868718	5.620550038
279.	Slc16a3	6.945050863	6.426333884	5.836187231	4.437822088
280.	Id3	4.583487342	4.208089986	4.383052977	6.531492208
281.	Macf1	3.717958719	3.60884626	4.029532151	5.915345592
282.	Gm766	7.995416072	7.811264388	7.455025028	5.643882783
283.	Scarb1	5.591773631	6.828275077	4.621913002	4.502783965
284.	Zg16	10.86516534	11.33160359	10.8307405	8.9068787
285.	Litaf	6.578078318	6.316466852	6.227059764	8.501657666
286.	Gm11048	9.236078311	9.861911075	11.51292392	11.1547272
287.	Pim1	5.063095489	4.826888824	4.81940828	7.034260772
288.	Krt4	2.884684169	2.849195706	2.978779212	5.039455058
289.	Rhod	4.284153999	3.559285399	4.452885389	6.090442082
290.	Tagln2	8.950785861	8.575156033	9.045549066	10.95402609
291.	Oas3	3.00420503	3.023245883	3.0840067	5.164493354
292.	Papss2	9.749187554	9.843105406	9.559719467	7.603445471
293.	Sis	10.81586134	11.35429443	11.11171875	9.013085096
294.	Gstm7	6.373675423	6.304236981	5.754883853	4.091835883
295.	Adh6a	8.275121645	8.630525142	8.127129351	6.267895124
296.	Cd36	5.394687451	5.505577332	4.093517877	3.311894957
297.	Tac1	6.362090593	6.413465155	5.64908834	4.142008929
298.	Areg	7.583898131	6.359487912	7.270985624	8.911743919
299.	Gm15293	7.205098377	7.788743736	9.444340922	9.074192225
300.	Steap1	3.715176308	3.604758954	4.130627918	5.877605612
301.	Ngfrap1	5.615720768	5.546528598	5.796543179	7.751172245
302.	Fbln1	7.144942639	7.433416429	6.620020878	5.068427573
303.	Ndufa4l2	5.251454669	3.924488192	3.216425579	2.870803536
304.	Cyp2u1	5.06823177	5.792989329	5.005364098	3.309666605
305.	Bmp8b	3.786211014	3.459829669	3.888852222	5.769719
306.	Cml1	5.894384033	6.241220425	5.532286636	3.887784552
307.	Usp18	3.006224965	3.035305049	2.962568463	5.080410394
308.	Tm4sf4	9.423781099	8.659819999	8.439585348	10.74153967
309.	Defa5	8.276581212	8.990563247	10.56611803	10.08989067
310.	Dok2	3.09901496	3.112977842	3.161451981	5.199934651
311.	Casp6	7.92426124	8.101708375	7.456314995	5.824265959
312.	Fcgbp	9.230467058	9.497510684	9.807155307	7.490580383
313.	Gm6665	8.82995676	8.654708454	7.723494667	6.576302936
314.	Suox	5.537061844	4.93010149	5.928819396	7.360787642
315.	Pmaip1	5.826654445	5.129629475	5.388369827	7.431685252
316.	Gm14850	10.15958581	10.74459058	12.36611259	11.96939344
317.	Ppp1r9a	3.202020808	3.21198506	3.463122969	5.338892347
318.	Gm10129	5.88520964	5.904942685	5.928435225	7.966113062
319.	Anxa5	5.64483256	4.867136977	5.754985156	7.314295959
320.	Cd302	6.142476661	6.349327291	5.952886067	4.129156764
321.	Sprr1a	8.344323608	6.726944057	6.803746424	8.691990531
322.	Rtp4	3.046138566	3.298963514	3.224605682	5.221173438
323.	Bche	7.671370908	7.560763353	7.373984632	5.510811564
324.	Ptpn21	4.134497241	3.786949389	4.24612057	6.055984348
325.	Chac1	5.37180636	4.655663286	5.461169333	7.068259562
326.	Cyp4f16	7.65438777	6.619664222	7.833260283	5.638050359
327.	Dppa5b	6.703215702	6.828545495	5.372517116	4.750717982
328.	Tm4sf5	10.41229437	10.55293429	9.911123671	8.33406249
329.	Samd9l	5.051344128	4.459322664	4.959492633	6.784743764
330.	Ephx2	8.107988383	8.251036176	7.224625288	6.050183841
331.	Btnl3	7.26698498	7.070214224	6.947784405	5.089807573
332.	Gadd45b	5.055135078	4.926146342	4.618591681	6.853961392
333.	Sh3bp5	3.397811903	3.407618857	3.667407603	5.496164212
334.	Cyp4f14	10.23063657	9.896228845	9.773142524	7.983570256
335.	Cyp2c38	5.111296586	5.177802298	4.072403182	3.039976419
336.	Lamc1	4.118203319	3.984943053	4.568586963	6.180034934
337.	F2rl1	5.71285353	5.262355948	5.994083706	7.582904498
338.	Cbr1	9.916459035	10.0027816	9.194745852	7.820624979
339.	Fahd1	7.855998123	8.020048653	7.348313251	5.808954824

340.	Tead1	4.052064531	4.080591413	4.470080209	6.178372507
341.	Ly6e	5.973908761	6.110877707	7.009710423	8.156252912
342.	Fam132a	5.950076645	6.374829755	4.706734082	4.251800946
343.	Myadm	6.355489017	6.079380909	6.425931613	8.269706145
344.	Slc44a4	6.369146071	6.006929778	6.968274884	8.287692875
345.	Gm14851	10.15412834	10.45317863	12.1963111	11.80422994
346.	Otop3	5.475011735	5.270357004	4.671850575	3.25891239
347.	Amica1	6.830956128	6.994425172	6.917808547	4.923147384
348.	AA467197	6.272033096	5.072340612	4.663446387	3.887403509
349.	Ces2c	9.177346335	9.020924635	8.978283798	7.076998406
350.	Tnks1bp1	5.567144256	4.991078985	5.626431271	7.298766792
351.	Bcam	3.440126695	3.272196713	3.359659268	5.34067725
352.	Gm13698	3.568183094	3.485153628	5.220682147	5.273278385
353.	Sepp1	8.104014289	8.322632956	7.052592892	6.176619428
354.	Wls	4.465072425	4.452666581	5.053396318	6.557695769
355.	Plec	5.526598586	5.168404725	5.472768934	7.344061551
356.	Kctd10	6.197395805	5.740639706	6.263979279	7.990018296
357.	Gstm2	6.703272452	6.674242682	5.93858761	4.591992786
358.	Rnf213	3.966782288	4.156451254	4.551488902	6.14150069
359.	Ccdc120	4.157572475	3.642516963	4.167629967	5.903628859
360.	Crip2	3.616443269	3.442594627	3.297110125	5.409766153
361.	Agrn	5.990953802	5.757419101	6.414132635	7.950850805
362.	Cyp2c65	9.063934801	8.997264508	7.942318621	6.987142126
363.	Gjb4	2.909710991	2.862641567	3.06537829	4.907788057
364.	X2210404O07Rik	11.16819754	11.47455485	10.73989353	9.253277054
365.	Cat	8.805104356	9.014889666	8.178767753	6.835070751
366.	Arhgap21	5.50264602	5.397068196	5.862196694	7.509796247
367.	Vdr	8.124346526	8.092614389	8.005420514	6.11361486
368.	Dmbt1	9.078403395	8.575486164	9.862299779	7.519168577
369.	Il1rn	5.313081642	3.478423707	4.945633847	5.735557878
370.	Rin1	4.424115816	3.55891167	4.29099029	5.899376196
371.	Gm7008	4.616384517	4.060130029	4.52658019	6.300485678
372.	Gm10639	10.81290721	10.91072297	9.597005228	8.885618499
373.	H2afv	7.686606647	7.769529771	7.180575301	5.655935185
374.	Gm10935	7.233411611	7.499577076	7.855054997	5.63763967
375.	Car4	4.249312841	4.883848153	5.559754134	3.258522906
376.	Fam189a2	3.556128129	3.174013689	3.643474548	5.365406434
377.	Hist1h2bc	7.558084801	7.599821138	6.31019868	5.616091754
378.	Tsc22d2	4.171645309	4.003443708	4.263551726	6.083613085
379.	Slc44a2	3.882690455	3.736849374	4.037052768	5.816759481
380.	Crlf3	4.233796711	4.067473611	4.433147547	6.165325334
381.	Fos	4.76917198	4.251659124	4.604417299	6.436055849
382.	Calcb	3.231649147	2.869359337	3.080090587	4.980354149
383.	Pak6	3.220790959	3.182422975	3.097701039	5.101821942
384.	Akr1c12	8.847150684	8.752394951	8.14222366	6.748892064
385.	Lats2	3.263102972	3.165001825	3.211147904	5.145893574
386.	Prap1	11.94814336	11.92484007	11.40265952	9.893311484
387.	Eif2s3y	4.45388022	4.581313928	2.849195706	2.849195706
388.	Fam83h	5.859803679	5.296823273	5.925344057	7.536282508
389.	Faah	6.580712193	6.791819636	6.378663335	4.686762435
390.	Cbr3	6.513432778	4.945112505	5.707290148	4.286778731
391.	Ces2d.ps	8.584987218	8.424599314	8.557227029	6.605155577
392.	Adh6.ps1	4.932010552	5.297193239	4.363119563	3.10280272
393.	E130012A19Rik	4.248268039	3.755241426	4.019184369	5.884205692
394.	Prkci	5.701682181	5.388639859	5.974663981	7.546560189
395.	Ostb	6.055933005	6.901681537	6.712042263	4.782814631
396.	Raph1	4.840820478	4.455092043	5.024814568	6.62072982
397.	Aim1	4.789380508	4.595594315	5.195620849	6.698506756
398.	Hck	5.194575332	5.673716279	3.927840422	3.724527041
399.	Slc39a5	8.087528295	8.285845451	7.946043173	6.223712931
400.	Pwwp2b	4.523734746	4.271232618	4.617923468	6.349858187
401.	Ces2b	5.546074099	5.458760655	5.780945292	3.715129027
402.	Aadac	9.122502926	9.271154008	8.408012424	7.190945163

403.	Ifrd1	5.375624091	4.822724426	5.281347022	6.993042587
404.	Sgk2	3.765281625	3.341556884	3.87825343	5.499868221
405.	Tgfbr2	5.15941209	4.772906892	5.217837885	6.903292558
406.	Chga	7.272346629	7.265539246	6.20679424	5.309409956
407.	Ddah1	4.341269158	4.262783156	4.606669156	6.272667961
408.	Aqp11	7.272962145	7.542527135	6.713581274	5.42149205
409.	Arhgap29	3.290395998	3.132521662	3.434711788	5.153707316
410.	Egln3	7.423354309	6.71489744	6.195447581	5.185920894
411.	Cnksr1	4.891149382	4.434876421	5.002905228	6.591612499
412.	Myo1e	5.421573027	5.016989585	5.625623291	7.16060184
413.	Ugt2a3	7.10750243	7.325735886	6.683693941	5.242307952
414.	Creb3l3	8.011239771	8.15207579	7.117403016	6.127692344
415.	D630039A03Rik	5.645013754	5.869662622	5.878898903	3.940915722
416.	Tspan4	3.798109929	3.668566661	4.157788775	5.697660253
417.	Hcn3	7.227175663	7.6931693	6.784763493	5.51982272
418.	Tff3	11.90004799	12.10191734	11.69538223	10.06375316
419.	Retsat	6.92934561	7.109879879	6.034380424	5.081468205
420.	Gm684	5.096816638	5.159531863	4.729313731	3.170235434
421.	Ptrh1	5.096402912	4.324323759	5.001344764	6.539834601
422.	Tns4	4.353020415	4.493727078	5.167532162	6.393673171
423.	Alox5ap	4.814993372	5.144946389	3.48580103	3.293791384
424.	Cyp2c69	4.991500762	5.080235002	4.121205146	3.083042369
425.	Ugt1a9	10.06831391	9.997382045	9.684900338	8.087301994
426.	Dhrs4	8.529725702	8.576747197	7.832448775	6.585147554
427.	Sult1d1	8.128802966	7.766422669	7.663388869	6.040258831
428.	Cdk6	3.912597852	3.892074472	4.870922145	5.838115392
429.	Gsta2	9.916096827	9.932133385	8.665135241	8.084369513
430.	Pik3cb	4.210012231	4.257148763	4.345638258	6.118234011
431.	Scin	7.783176599	7.901181808	7.901798123	6.016583664
432.	Aldh1b1	10.18714171	10.33016872	10.17539019	8.389005019
433.	Ces2f	6.823525344	6.819340418	6.789841282	4.964751502
434.	Tln1	5.253849831	5.126309993	5.67208248	7.134213336
435.	Oat	10.77265679	11.15539691	10.73504997	9.088879139
436.	Cml5	7.2674484	7.122699176	6.393542905	5.258067413
437.	Plekhg3	4.909740953	4.47957087	5.060719855	6.579590752
438.	Cyp2c66	9.640954222	9.492663764	8.722601604	7.642498844
439.	Spink4	11.00667789	11.32105979	10.97234503	9.29805206
440.	Pacsin2	7.068033973	6.904148989	7.146526043	8.857285339
441.	Dusp3	4.050991434	3.659725512	3.882264362	5.663907442
442.	Gsta1	10.96880116	11.06487036	9.935239043	9.143424206
443.	Ugt1a8	10.02314431	9.952229084	9.669901482	8.081610796
444.	Otud7b	4.825862731	4.488577358	4.883715318	6.524337598
445.	Gm11437	5.89969857	5.748058918	5.658668432	3.954654438
446.	Fmo5	7.308278627	7.130785016	7.28235075	5.423242513
447.	Slco2b1	5.340599936	5.581845245	5.228523389	3.584455384
448.	Ugt1a6b	9.887959059	9.827519697	9.540114811	7.956483488
449.	Gm7849	7.29299063	8.087543959	9.330095853	8.944826631
450.	Ugt1a10	9.957145434	9.897803098	9.613243014	8.028269936
451.	Ugt1a5	9.992191136	9.935453755	9.650266291	8.064890466
452.	Ugt1a2	9.987408624	9.930654226	9.64521447	8.060184768
453.	Gsn	7.099708206	6.875910604	7.163703919	8.848304694
454.	Ugt1a1	10.15116443	10.10415636	9.812369341	8.229289394
455.	Hnf4g	8.393893904	8.355041358	7.956793273	6.461956611
456.	Ccl20	4.251936733	3.8700017	4.195820587	5.889732284
457.	Csrnp1	3.400669353	3.340502925	3.281150781	5.151385061
458.	Ace	6.812220303	6.956361475	6.197978396	4.966466274
459.	Ifit3	3.001788144	2.951802259	2.981026507	4.788438032
460.	Slc2a2	7.26738792	7.625059982	7.414973121	5.652402585
461.	Ifi27l1	4.83973023	4.803386653	4.626589418	6.554341889
462.	Car2	4.350086657	3.837518188	4.123377004	5.86161505
463.	Actb	10.7448806	10.54917341	10.62736344	12.44030907
464.	Pycard	8.52915138	8.315380688	8.524058105	6.660977768
465.	Prrg4	3.808985661	3.434656534	3.960623756	5.479769146

466.	Itgb6	3.668111623	3.379860142	3.693335559	5.355980733
467.	Slc13a2	5.677260756	6.500065425	5.532333851	4.320006584
468.	Ereg	4.364837975	3.760389981	4.666583516	5.896084391
469.	Npnt	3.330651037	3.151397031	3.326359444	5.055346681
470.	Stbd1	3.309690535	2.984160606	3.16793423	4.926838865
471.	Cdc42se2	6.43275176	6.239116177	6.510730154	8.168625882
472.	Ddc	7.493801808	7.264303078	6.941970417	5.502685355
473.	Reg3g	8.517959811	7.962072822	8.431075037	6.583207146
474.	Ugt1a6a	8.645409186	8.603484934	8.315530539	6.758208958
475.	Phlda2	4.820946134	4.272116889	4.680484221	6.316000987
476.	Arf2	4.331848978	4.195325872	4.533055692	6.117826559
477.	Dhrs3	5.486389641	5.615866006	5.237786152	3.694652096
478.	Ms4a10	8.961362257	9.022602388	8.007405723	7.147853426
479.	Mgst2	8.306173444	8.320534502	7.973657835	6.452896868
480.	Pcyt1a	6.10804204	5.47640877	6.062137764	7.560740711
481.	Oasl1	5.658432412	5.007980088	5.515476031	7.078005204
482.	Pear1	3.528880742	3.217063845	3.617812099	5.194820979
483.	Slamf9	3.817860208	3.378682966	3.646254689	5.343335418
484.	Acot9	4.887188405	4.457587186	4.563796272	6.361925973
485.	Endod1	4.425825303	4.281511743	4.68810914	6.195580912
486.	Oas1a	4.133114963	4.435353776	4.426118519	6.071762937
487.	Scp2	7.172661378	7.311719365	6.454400528	5.387413435
488.	Adk	6.757032258	6.744685602	6.888073454	8.549306258
489.	Pink1	7.033095969	7.217335058	6.404054865	5.275811204
490.	Adamts15	3.663399229	3.256945002	3.713582522	5.247120635
491.	Hepacam2	6.035502857	6.191444871	6.019128327	4.33809812
492.	X4930572J05Rik	4.29840201	4.238577237	4.553689314	6.08735112
493.	Gm15292	7.925170124	8.978106181	9.936864664	9.532374632
494.	Ugt1a7c	9.79186307	9.788111932	9.552119237	7.980992238
495.	Dusp9	3.265873247	3.125873088	3.385075611	4.989565535
496.	S100a16	6.418263556	5.592033643	6.31432222	7.688160393
497.	Hadh	8.420299371	8.599416798	8.013523948	6.676127043
498.	Ptgr1	8.279292084	8.070731498	7.26583388	6.370644862
499.	Acot13	7.999465972	7.900525961	6.808826533	6.211332723
500.	Slc22a1	6.249018126	6.575954562	6.290674529	4.662190763

© 2020 by the authors. Licensee MDPI, Basel, Switzerland. This article is an open access article distributed under the terms and conditions of the Creative Commons Attribution (CC BY) license (http://creativecommons.org/licenses/by/4.0/).
